# Herpesvirus Screening in Childhood Hematopoietic Transplant Reveals High Systemic Inflammation in Episodes of Multiple Viral Detection and an EBV Association with Elevated IL-1β, IL-8 and Graft-Versus-Host Disease

**DOI:** 10.3390/microorganisms10081685

**Published:** 2022-08-22

**Authors:** Moisés H. Rojas-Rechy, Félix Gaytán-Morales, Yessica Sánchez-Ponce, Iván Castorena-Villa, Briceida López-Martínez, Israel Parra-Ortega, María C. Escamilla-Núñez, Alfonso Méndez-Tenorio, Ericka N. Pompa-Mera, Gustavo U. Martinez-Ruiz, Ezequiel M. Fuentes-Pananá, Abigail Morales-Sánchez

**Affiliations:** 1 Research Unit in Virology and Cancer, Children’s Hospital of Mexico Federico Gomez, Mexico City 06720, Mexico; 2 Postgraduate Program in Biomedicine and Molecular Biotechnology, National Polytechnic Institute, Mexico City 11340, Mexico; 3 Bone Marrow Transplant Service, Children’s Hospital of Mexico Federico Gomez, Mexico City 06720, Mexico; 4 Sub-Direction of Diagnostic Auxiliary Services, Children’s Hospital of Mexico Federico Gomez, Mexico City 06720, Mexico; 5 Department of Clinical Laboratory, Children’s Hospital of Mexico Federico Gomez, Mexico City 06720, Mexico; 6 National Institute of Public Health, Cuernavaca 62100, Mexico; 7 Laboratorio de Biotecnología y Bioinformática Genómica, Escuela Nacional de Ciencias Biológicas, Instituto Politécnico Nacional, Mexico City 11340, Mexico; 8 Infectious and Parasitic Disease Medical Research Unit (UIMEIP), Pediatric Hospital in National Medical Center (CMN-SIGLO XXI), Mexican Institute of Social Security (IMSS), Mexico City 06720, Mexico; 9 División de Investigación, Facultad de Medicina, Universidad Nacional Autónoma de México, Mexico City 04510, Mexico; 10 Laboratorio de Investigación en Patología Experimental, Children’s Hospital of Mexico Federico Gomez, Mexico City 06720, Mexico

**Keywords:** hematopoietic stem cell transplant, herpesvirus, EBV, HCMV, HHV6, HHV7, systemic inflammation, graft-versus-host disease

## Abstract

Infections remain a major cause of morbidity and mortality among hematopoietic stem cell transplant (HSCT) recipients. Unlike Epstein–Barr Virus (EBV) and Human Cytomegalovirus (HCMV), Human Herpesvirus (HHV) 6, HHV7 and HHV8 are not routinely monitored in many centers, especially in the pediatric population of low–medium income countries. We screened EBV, HCMV, HHV6, HHV7 and HHV8 in 412 leukocytes-plasma paired samples from 40 pediatric patients assisted in a tertiary hospital in Mexico. Thirty-two underwent allo-HSCT, whereas eight received auto-HSCT. Overall viral detection frequencies in allo- and auto-HSCT were: EBV = 43.7% and 30.0%, HCMV = 5.0% and 6.7%, HHV6 = 7.9% and 20.0% and HHV7 = 9.7% and 23.3%. HHV8 was not detected in any sample. Interestingly, HHV6 and HHV7 were more frequent in auto-HSCT, and HHV6 was observed in all episodes of multiple detection in auto-HSCT patients. We found EBV DNA in plasma samples, whereas HCMV, HHV6 and HHV7 DNA were predominantly observed in leukocytes, indicative of their expansion in cellular compartments. We also found that IL-1β, IL-2, IL-6 and IL-8 were significantly increased in episodes in which multiple viruses were simultaneously detected, and samples positive for EBV DNA and graft-versus-host disease had a further increase of IL-1β and IL-8. In conclusion, the EBV, HCMV, HHV6 and HHV7 burdens were frequently detected in allo- and auto-HSCT, and their presence associated with systemic inflammation.

## 1. Introduction

Hematopoietic stem and progenitor cell transplantation (HSCT) has become a standard treatment for hematological and non-hematological diseases with no other curative alternative [1]. Graft-versus-host disease (GvHD) is a frequent multi-organ, life-threatening complication of patients undergoing allogeneic (allo)-HSCT. Allograft recipients experience a severe risk of infection given the high doses of immunosuppressant drugs they receive to prevent and treat GvHD [2]. In GvHD, donor T-cells recognize and attack antigen-bearing cells, generating symptomatic organ damage. Moreover, patients undergoing allo- and autologous (auto)-HSCT are also immune-compromised because they usually receive potent chemotherapy to treat the primary disease [1]. Since cancer is often the underlying disease of these patients, a competent immune system is central to a good donor versus cancer cell response [3]. Due to the increasing numbers of HSCTs in the world, it is urgent to dissect one from the other and recognize an increased risk of developing GvDH, but today, we are still unable to recognize protective from pathogenic immunity.

One of the most critical challenges for HSCT recipients is the occurrence of viral infections caused by human herpesviruses (HHV) [4]. Herpesviruses are among the most prevalent infections in humans, usually starting at an early age and persisting for the lifetime of the infected individual. Each herpesvirus infects from 70–100% of adults worldwide, with most children chronically carrying up to seven of these viruses. Infection is usually asymptomatic, but there are clinical settings, such as immunosuppression, in which these viruses can be reactivated and associated with disease [5]. Indeed, beyond a clinical setting, peripheral blood viral loads of these viruses are usually undetectable.

Human cytomegalovirus (HCMV) and Epstein–Barr virus (EBV) are the most studied viruses in a transplantation setting and are known to cause end-organ disease and post-transplant lymphoproliferative disease, respectively. In addition, EBV and HCMV reactivations are associated with a poorer prognosis in adult patients with HSCT [2,3,4]. The incidence and implications of HHV6 and HHV7 reactivation have been less studied. For HHV6, complications range from mild fever and rash to encephalitis and central nervous system-related problems [6,7,8]. In general, both HHV6 and HHV7 are not routinely monitored in underdeveloped and developing countries, and their prevalence in HSCT and potential contribution to GvHD are less documented. In this study, we recruited pediatric patients who received allo-HSCT (n = 32) or auto-HSCT (n = 8), and screened EBV, HCMV, HHV6, HHV7 and HHV8 in paired leukocytes-plasma samples by multiplex qPCR. In plasma samples, we also evaluated the levels of a set of inflammatory cytokines.

## 2. Materials and Methods

### 2.1. Ethical Statement

This study (protocol numbers HIM/2016/021 SSA 1237 and HIM/2017/145 SSA 1469) was approved by the Ethical, Biosecurity and Scientific Review Boards of the Children’s Hospital of Mexico “Federico Gomez”. Parents/custodians, healthy controls (young adults) and children older than ten years of age willing to participate in the protocol signed a consent letter. Ten-year-old and younger children willing to participate in the protocol signed an assent letter. Every blood sample used in this protocol was taken only under the treating physician’s approval. The study was conducted according to the guidelines of the Declaration of Helsinki.

### 2.2. Patients and Clinical Samples

This study recruited pediatric patients receiving HSCT from June 2016 to April 2019. During this period, 49 children received HSCT at our institute. Of them, 46 were willing to participate. However, five were eliminated because they presented primary disease relapse during the first-month post-transplantation. In total, we included 40 pediatric patients that were followed for up to 365 days (Table 1). The study follow-up was constrained to the patients’ clinical follow-up and treatment adherence. Follow-up and sampling frequency are described in Table 1. Pre-transplant serology from some donors and patients can be found in Supplementary Table S1, but we were not able to collect all the data. Clinical and demographic data were extracted from medical records and retrospectively analyzed (Table 1). Patients received acyclovir as a prophylactic treatment to prevent complications from HCMV and HSV-1, at a dose of 1500 mg/m^2^/day for children weighing 40 Kg or more, and 750 mg/m^2^/day for children less than 40 Kg. We also included remaining blood samples from 15 healthy donors who attended the Blood Bank at our institute to donate.

### 2.3. DNA Isolation

We collected 1–4 mL of peripheral blood in tubes added with EDTA (BD Vacutainer). Samples were centrifugated at 3500 rpm for 15 min at 4 °C to separate the plasma from the cell pellet. The plasma was aliquoted in fresh tubes and maintained at −80 °C until use. The cell pellet was resuspended in PBS 1x. Trypan blue exclusion was used to assess cell viability. Cells were stored in lysis buffer (EL buffer, QIAGEN) at −80 °C until use. We isolated DNA from 200 μL of plasma or 1–4 × 10^6^ leukocytes using the QIAmp 96 DNA Blood kit (QIAGEN) and the QIAmp DNA Mini Kit (QIAGEN), respectively. In a few samples with poor cellularity, less than 1 × 10^6^ leukocytes were used for DNA extraction (Supplementary Table S2). DNA concentration and purity were evaluated by spectrophotometry with the Nanodrop One-C (Thermo Fisher Scientific, Waltham, MA, USA). For the viral screening, leukocytes and plasma specimens were analyzed separately.

### 2.4. Viral Detection

We previously standardized a two-tube multiplex qPCR for simultaneous detection of EBV, HCMV, HHV6 (with no distinction of HHV6A and HHV6B), HHV7 and the Kaposi-associated herpes virus KSHV/HHV8 [9]. Briefly, viral gene fragments cloned into commercially available plasmids were used to construct standard curves. The multiplex reactions were as sensitive and specific as simplex reactions within a dynamic range of at least six orders of magnitude (10^1^ to 10^6^ DNA copies). The limit of DNA detection (copy number) with a 95% confidence for each virus was: EBV = 21, HCMV = 18, HHV6 = 25, HHV7 = 21 and KSHV = 18. High efficiency and predictability were also assessed. PCR was performed in 20 µL final reaction using Quantitect PCR kit (QIAGEN). Each patient sample was run in three replicates. DNA quality was assessed by amplification of the beta-actin (*ACTB*) gene. Samples with no amplification of *ACTB* were eliminated. Controls with no DNA were routinely run.

### 2.5. Cytokine Detection

Plasma levels of G-CSF, GM-CSF, IFN-γ, IL-1β, IL-2, IL-4, IL-5, IL-6, IL-7, IL-8, IL-10, IL-12, IL-13, IL-17, MCP-1, MIP-1β and TNFα were measured in 138 (33.5%) plasma samples using the commercial kit Bio-Plex Pro Human Cytokine 17 (BIO-RAD, Hercules, CA, USA) in the Bio-Plex 200 Systems according to the manufacturer instructions. Concentrations were expressed as Log 10 of the measure in pg/mL of plasma.

### 2.6. Sanger Sequencing

We used Sanger sequencing to confirm the viral identity in 10% of the positive samples (single detection and multiple detection). PCR products were purified using the QIAquick PCR Purification Kit (QIAGEN). Forward and reverse strands were sequenced in the 3500 series genetic analyzer (ThermoFisher Scientific, Waltham, MA, USA) at the National Institute of Respiratory Diseases (Mexico City, Mexico). Alignments were performed using the program Unipro UGENE version 33.0 (macOS) with accession numbers EBV: NC_007605.1, HCMV: ON119199.1, HHV6: MF511176.2 and HHV7: NC_001716.2.

### 2.7. Statistical Analysis

We used the proportion test to compare differences in frequencies. Wilcoxon-matched pairs test was used for paired comparisons. For multigroup analysis, we performed the Kruskal–Wallis Test and Dunn multiple comparison test as post-hoc analysis. Survival analyses were performed by the Kaplan–Meier method. For all the analyses, a *p* < 0.05 was considered significant. We used Prism version 9.4 (macOS) for making plots and analyzing data.

## 3. Results

### 3.1. EBV, HCMV, HHV6 and HHV7 DNA Are Detected in Allogeneic and Autologous HSCT at Different Frequencies but with a Similar Burden

We analyzed 40 pediatric patients who received HSCT. Those receiving allo-HSCT were followed for up to one year, whereas those receiving auto-HSCT were followed for up to 6 months (Table 1). We collected blood samples starting on day 7 pre-transplant and then periodically with a median of 14 and 19 days for allo- and auto-HSCT, respectively. In total, we analyzed leukocytes and plasma from 412 blood samples separately. We used multiplex qPCR (see Methods and ref. [9]) to quantify the viral load of EBV, HCMV, HHV6, HHV7 and HHV8/KSHV. These viruses are generally undetectable in peripheral blood in healthy immunocompetent hosts. Because the median age of the patients included in this study, the detection of viruses most probably reflects viral reactivation episodes in which the level of viral particles becomes detectable. We also included 15 samples from young adults who attended the hospital as volunteers to donate blood.

**Figure 1:**
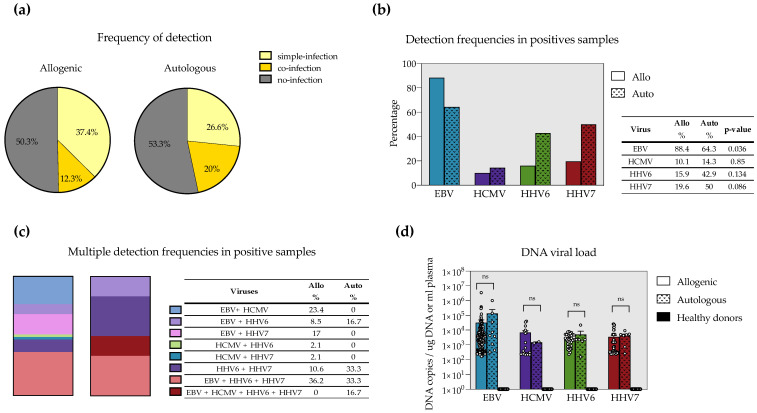
Frequency of single and multiple detection of EBV, HCMV, HHV6 and HHV7 in childhood allogeneic (allo) and autologous (auto) hematopoietic stem cell transplant (HSCT). Comparison of detection status (**a**), overall detection (**b**) and multiple detection (**c**) episodes between allo- and auto-HSCT. In (**b**), frequencies were compared by the Test of Difference in proportions. (**d**) Magnitude of the viral load seen for each virus in allo-, auto-HSCT and healthy donors. Mann–Whitney’s test was used to compare allo- versus auto-HSCT.

HHV8 was not detected in any patient, and it was not plotted in any figure. Thirty patients had at least one virus-positive sample during the follow-up. We observed 205 positive samples for at least one virus. The frequency of viral positivity, single detection and multiple detection were nearly the same for allo- and auto-HSCT (Figure 1a). Viral frequencies per virus are depicted in Table 1. EBV was the most frequent virus. Interestingly, HHV6 and HHV7 were more common in auto-HSCT than in allo-HSCT. HCMV was rarely detected. Whether this is due to the prophylactic treatment with acyclovir and whether this treatment differentially affects the detection of these viruses should be investigated in the future. In Figure 1b, we compared virus frequencies only in positive samples. EBV was significantly higher in allo-HSCT than in auto-HSCT. On the other hand, HHV6 and HHV7 frequencies were higher in auto-HSCT but with no statical significance, likely due to the low number of samples in auto-HSCT.

We next analyzed multiple detections. Overall, detection episodes with more than one herpesvirus were detected in 12.3% of the samples in allo-HSCT and 20% in auto-HSCT (Figure 1a). Nevertheless, fewer combinations of multiple detections were detected in auto-HSCT, whereas higher diversity of multiple detections was observed in allo-HSCT (Figure 1c). This was not surprising given the lower number of samples we had in auto-HSCT. Despite that, a still interesting observation is that all (100%) of multiple detection episodes in auto-HSCT involved HHV6. On the other hand, most (95.7%) of the multiple detection episodes seen in allo-HSCT included EBV. Interestingly, EBV+HHV6+HHV7 multiple detection was the most frequent regardless of the type of transplant. The low number of samples in auto-HSCT precluded doing statistical analysis.

Finally, we looked at the viral burden in the different types of transplants. EBV had the highest viral loads. Surprisingly, no differences in viral loads were observed between allo- and auto-HSCT for all the viruses (Figure 1d). In this analysis, we also included 15 samples from healthy, young adults in whom no viral load was detected.

### 3.2. EBV Is Mostly Found in the Acelluar Fraction, Whereas HCMV, HHV6 and HHV7 Predominantly Expand in the Cellular Compartment

**Figure 2:**
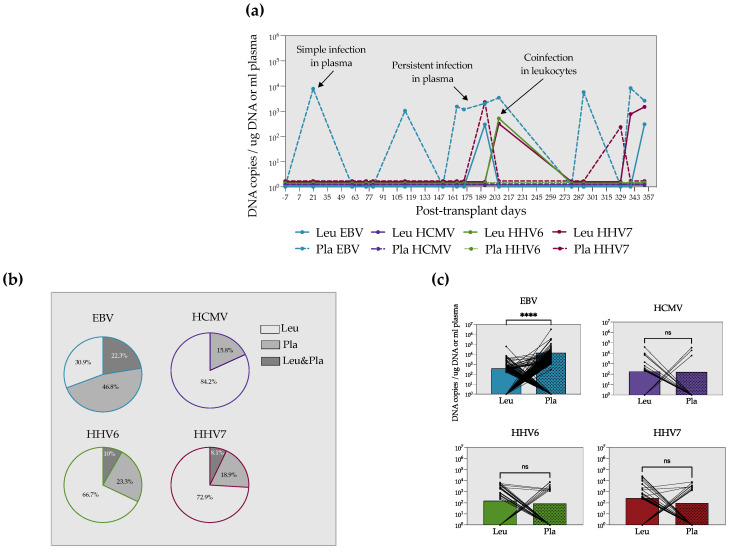
EBV DNA is found mainly in plasma, whereas HCMV, HHV6 and HHV7 DNA are predominantly in leukocytes. (**a**) Example of the detection episodes in plasma and leukocytes in a patient with allo-HSCT during one year of follow-up. (**b**) Frequencies of EBV, HCMV, HHV6 and HHV7 detection in leukocytes, plasma or leukocytes and plasma simultaneously. (**c**) Paired analyses of the viral load in leukocytes and plasma for each virus. The mean value is up to the bars. Paired Mann–Whitney test. **** = *p* < 0.0001, ns = non-significant.

We independently analyzed the viral load in leukocytes and plasma. Given the low number of samples of auto-HSCT we collected, we only included patients with allo-HSCT for this analysis. Figure 2a shows the distribution of detections in plasma and leukocytes during one year of follow-up of one patient with allo-HSCT as an example. Detection episodes in plasma and leukocytes for the rest of the patients can be found in Supplementary Figure S1.

Only 0–22.3% of the samples were simultaneously positive in leukocytes and plasma (Figure 2b). Interestingly, for HCMV, HHV6 and HHV7, the majority of positive samples were found in leukocytes (Figure 2b). On the contrary, EBV DNA was more frequently found in plasma (47%) than in cells. Furthermore, in a paired comparison of the viral load in the two specimens, we observed that EBV DNA copies were significantly higher in plasma. We did not see differences between plasma and leukocyte viral loads for the other herpesviruses (Figure 2c).

### 3.3. Allo-HSCT Recipients Show Increased Levels of IL-1β, IL-2, IL-6 and IL-8 during Multiple Detection Episodes

It has been shown that allograft inflammation contributes to graft rejection in solid organ transplantation [10]. To investigate the correlation between herpesvirus detection and systemic inflammation, we evaluated the plasma levels of 17 cytokines (see material and methods).

**Figure 3:**
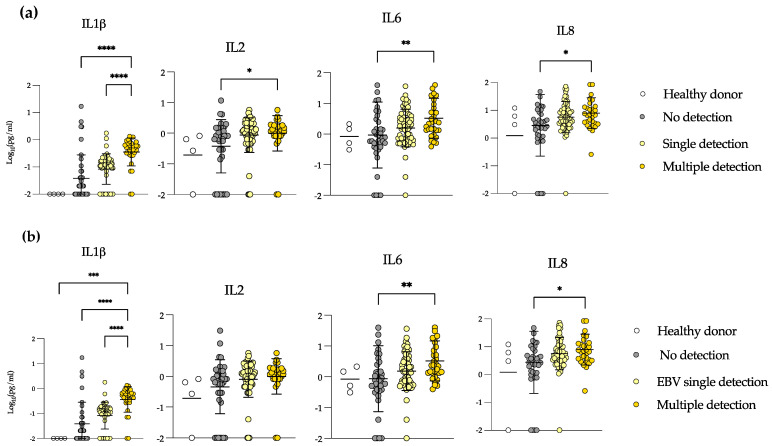
Samples in which multiple viruses were detected show high systemic levels of inflammatory cytokines. Plasma levels of IL-1β, IL-2, IL-6 and IL-8 in healthy donors, no viral detection, single detection ((**a**): any virus, (**b**): EBV) and multiple detection samples in patients with allogeneic transplant. Mann–Whitney test. * = *p* < 0.05, ** = *p* < 0.01, *** = *p* < 0.001, **** = *p* < 0.0001.

We compared samples according to their viral detection status as non-detected, single detection (any virus) and multiple detection (any double or triple detection) and included healthy donor samples as controls. We found that IL-1β, IL-2, IL-6 and IL-8 were significantly higher in multiple detection than in non-detection samples (Figure 3a). In addition, IL-1β was significantly higher in multiple detection than in single detection samples. We did not see differences in the other measured cytokines (not shown).

The majority of the samples in allo-HSCT were EBV-positive; we specifically addressed the levels of those same cytokines in EBV-single detection vs. samples in which EBV was detected with other viruses (Figure 3b). We saw the same results for IL-1β, IL-6 and IL-8. IL-1β levels increased from non-detection to EBV-single detection samples, and significantly rose in multiple detection samples. IL-6 and IL-8 levels were also significantly higher in samples with multiple detection than in samples with no detection. These results demonstrate that in multiple detection episodes, there is a systemic inflammation evidenced by high levels of IL-1β, IL-2, IL-6 and IL-8. In addition, EBV detection is the one more closely correlating with the enhanced levels of these cytokines, and at the same time, EBV reactivation may be an important driver and may have an impact on GvHD or vice versa. However, directionality cannot be determined.

### 3.4. Elevated Levels of IL-1β and IL-8 in EBV-Positive Samples during GvHD

**Figure 4:**
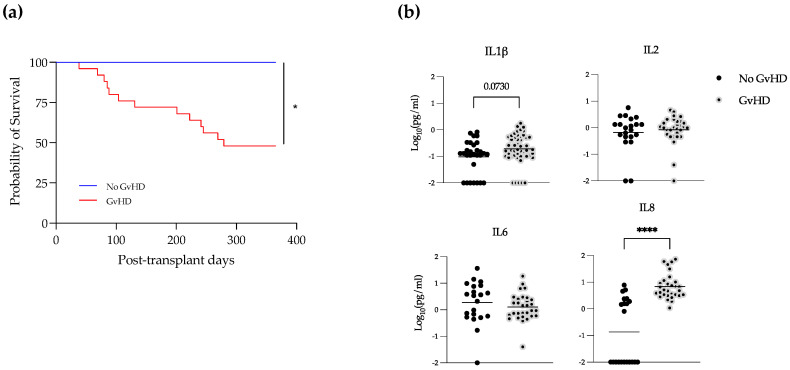
IL-1β and IL-8 increase in EBV-positive samples during GvHD. (**a**) Survival curve of patients with or without GvHD. Kaplan–Meier method, * = *p* < 0.05. (**b**) Plasma levels of the indicated cytokines in samples EBV-positive with or without GvHD. Mann–Whitney test., **** = *p* < 0.0001.

In these patients, GvHD was significantly associated with poor survival (Figure 4a). We looked at the levels of cytokines in allo-recipients with or without GvHD and subclassified GVHD patients in alive or dead, but no statistical differences were found between groups (not shown). We then analyzed levels of cytokines in EBV-positive samples, comparing patients who developed GvHD or not during their follow-up. Interestingly, we saw that IL-8 was significantly increased in recipients suffering GvHD (Figure 4b). In addition, there was a trend towards an increase in the levels of IL-1β in GvHD and EBV double-positive patients.

## 4. Discussion

Infections remain a significant cause of morbidity and mortality in HSCT. Recipients of allo-HSCT are given high doses of immunosuppressants to prevent or treat GvHD. Conversely, upon auto-HSCT, patients do not receive immunosuppressant drugs. Still, very high doses of chemotherapy to eliminate tumors can suppress or even deplete the bone marrow [11]. In the pre-engraftment period, gastrointestinal mucositis, neutropenia, and the use of catheters constitute additional risk factors for infection in HSCT [12]. Infection complications are therefore present in recipients of both allo- and auto-HSCT. Nevertheless, the frequency and severity of infection-associated diseases can differ between the two transplant schemas. For example, fungal infection occurrence is lower than 1–2% in auto-HSCT [13,14,15]. However, the incidence of invasive fungal infections has been reported to be between 6 to 22% in allo-HSCT [16,17,18].

In this cohort of patients, EBV was the most frequently detected virus (allo-HSCT = 43.7% and auto-HSCT = 30.0%). Other authors have reported frequencies of 14.6% [19] and 42.6% [20] in allo-HSCT. The rates of herpesvirus detection may result from several factors, such as the level and type of immunosuppressant, the underlying disease, impaired lymphocyte reconstitution, the sensitivity of the diagnostic assay, or the many factors that influence herpesvirus reactivation [21]. For instance, graft T-cell depletion, the use of anti-lymphocyte serum and the use of anti-CD3 monoclonal antibodies have been tested for the prevention or treatment of GvHD [22]. However, these treatments have been shown to be risk factors for EBV-associated PTLD in HSCT patients [23]. Bonong et al. performed a meta-analysis that included 77 studies, and observed that these associations were unclear, as there were contradictory results. According to this meta-analysis, the only treatment consistently associated with enhanced EBV burden and PTLD was anti-thymocyte immunoglobulin [24].

HCMV had a low frequency of detection in both allo- (5.0%) and auto-(6.7%) HSCT in our analysis. Other studies reported similar loads in both transplant schemes when prophylactic therapy was given to patients [4,25]. In the literature, HCMV detection ranges from 28 to 88%. It has been associated with an increased risk of overall mortality and non-relapse mortality [26].

Although auto-HSCT usually involves shorter or less patients’ hospitalizations, we observed that the frequencies of detection of HHV6 and HHV7 were most frequent in the first 100 post-transplant days in the auto- HSCT than in the allo-HSCT (20 vs. 7.9% and 23.3 vs. 9.7%, respectively). This is interesting since the immune reconstitution in auto-HSCT is faster. Nevertheless, the number of patients/samples is uneven between the two types of transplants and then not enough to make a conclusion. HHV6 studies also observed a wide range of detection frequencies, from 13.9% to 93.6% [27]. According to a meta-analysis, reactivation of this virus is associated with GvHD [27]. Finally, HHV7 had reported frequencies going from 8.6% to 60%, with a weak association with encephalitis [28,29], severe GvHD and sepsis secondary to severe immunosuppression.

We did not observe an association between detection (either single or multiple) and GvHD perhaps because of the number of samples tested or the follow-up time. Interestingly, we found that HHV6 was present in all multiple detection episodes in auto-HSCT, i.e., patients who were only followed for six months. Another study also reported HHV6 as the most frequently detected virus [4]. The most frequently observed viruses in simple detection maintained their frequency in multiple detections. Unlike EBV and HCMV, HHV6 and HHV7 are not routinely screened in many centers [30]. Our data and the studies mentioned above support that HHV6 and HHV7 should be included in the routine testing of transplanted patients with hematopoietic stem cells and solid organs. Indeed, other studies have shown an association between multiple detection and clinical symptoms—for instance, EBV and HCMV co-detection with lower one-year overall survival and lower one-year leukemia-free survival [10] and with increased PTLD risk [31]. Multiple detections of HCMV, HHV6 and HHV7 has also been associated with HCMV syndrome, hyperbilirubinemia and thrombocytopenia [32].

The utility of some interleukins as biomarkers, in guiding clinical therapy for patients with GvHD has been the source of considerable interest [33]. In this regard, we also tested a wide array of Th1/Th2/Th17 cytokines together with a few chemokines influencing myeloid cell function. We observed an increase in the levels of IL-1β, IL-2, IL-6 and IL-8 correlating with multiple herpesvirus detection. In addition, the samples positive for EBV DNA and for GvHD had a further increase in the levels of IL-1β and IL-8. Whether EBV is the cause or consequence of the increased cytokine levels and the correlation with GvHD is not known. We think that it is interesting that EBV DNA was mainly detected in plasma and that the viral load was significantly higher in plasma than in leukocytes, contrary to the other viruses tested, which together may support potential mechanisms of viral reactivation and/or cellular-death-driven inflammation. Tanaka J. et al. observed that peripheral blood mononuclear cells isolated from GvHD patients expressed high levels of IL-6 mRNA [34], and IL-6 has been consistently found elevated in chronic GvHD [35], and has been recognized as one of the participants of the cytokine storm involved in hyper-acute GvHD [36]. The use of Ruxolitinib, a JAK inhibitor that results in diminished IL-6 expression, has been proposed to treat GvHD. In a clinical study, 85% of patients responded to Ruxolitinib and the six-month overall survival was 97.4% [37] (ClinicalTrials.gov number, NCT03112603). On the contrary, low doses of IL-2 have been shown to protect patients and mice in experimental models of HSCT against GvHD [38]. IL-2 is an important cytokine for the development of CD4 T-cells, including immune suppressive Tregs [39], and low absolute numbers of Tregs characterize patients with GvHD. Low-dose IL-2 enhanced Treg numbers and alleviated symptoms associated with GvHD [40].

Tanaka J. et al. also found elevated IL-1β transcription in PBMCs from GvHD patients [34]. Single nucleotide polymorphisms and variable number tandem repeats in the IL-1β promoter that drive higher expression of this cytokine have been shown to increase the risk for GvHD [41]. In a mouse model of GvHD an inhibitor of the IL-1 receptor prevented the appearance of GvHD [42]. However, in a clinical double-blinded, placebo-controlled randomized trial, the use of IL-1β receptor antagonists did not prevent the development of GvHD [43]. Since IL-1β is also a cytokine that participates in the homeostasis of tissues, [41] the use of antagonists may be toxic and complicate the picture of a potential prophylactic or therapeutic effect. The JAK inhibitor Ruxolitinib also prevents the expression of IL-8 [44], and elevated IL-8 correlates with the risk of GvHD [45,46]. Both IL-1β and IL-8 have been found elevated in EBV-positive nasopharyngeal carcinoma (NPC), and polymorphisms in IL-1β correlate with the risk of this disease in endemic regions [47,48]. Moreover, elevated IL-8 is characteristic of multiple EBV-associated diseases, such as NPC, gastric cancer, hemophagocytosis, chronic periodontitis, multiple sclerosis, murine models of NK/T lymphoma, infectious mononucleosis (IM), and post-IM chronic fatigue syndrome [49,50,51,52,53,54]. Multiple EBV gene products have been reported as responsible for IL-8 up-regulation, such as LMP1, EBER2, RPMS1 and BZLF1 [51,55,56,57,58]. Particularly, in NPC, IL-8 levels correlate with tumor invasion and the patient´s prognosis [53,58,59,60], and a decreased EBV load and decreased levels of IL-8 correlate with an NPC-positive response to treatment [61,62]. The mRNA expression of IL-8 has been widely used as a marker of inflammation during endothelial cell activation [63]. Notably, endothelial cell damage may trigger the initiation of GvHD [64]. In addition, the vascular endothelium can be a target of GvHD in the early phase and circulating endothelial cells represent surrogate markers of endothelial damage [65]. The timing of IL-8 production in allo-HSCT patients deserves more investigation to determine the role of IL-8 production in the context of GvHD.

In conclusion, in this study, we consistently observed elevated viral loads of herpesviruses, especially EBV alone or together with HHV6 and HHV7. Although these detection peaks were not associated with GvHD, evidence of a correlation with increased systemic inflammation was found. In particular, samples with both EBV detection and elevated IL-1β and IL-8 were enriched in GvHD. These data further support the screening of EBV and HCMV, but also of HHV6 and HHV7, for the prevention of GvHD in pediatric patients with HSCT.

## 5. Study Limitations

There are several limitations to this study. It is an observational-correlative study that only determines associations without establishing any directionality between the associated variables. In this case, it was not possible to conclude whether the appearance of inflammation and GvHD is a cause or a consequence of the enhanced viral detection, mainly of EBV. The correlation only establishes an association between different variables.

Furthermore, we were not able to establish relevant clinical thresholds for the observed viral loads of the various herpesviruses and the clinical assessments. Viral loads did not significantly change in samples with single vs. multiple virus detections. We were unable to associate a clinical measurement with either plasma or leukocyte-associated loads, arguing for complex interactions between the viral life cycle and the hosts, even in largely immunosuppressed host. Still, it is important to consider that despite the ubiquity of herpesviruses in the world population, viral DNA is generally undetectable in healthy carriers. The sole detection of viral genomes does not necessarily imply a disadvantageous clinical outcome. Thresholds of clinical-medical relevance must be established to predict or diagnose associated diseases. But these thresholds are not straightforward to predict and are highly heterogeneous among different authors. Moreover, they are mostly proposed to predict the development of post-transplant lymphoproliferative disease (PTLD) [66,67,68,69,70,71,72,73,74,75].

The Children’s Hospital of Mexico is one of the main pediatric transplant centers, and the doctors have their own clinical intervention criteria, based on the Fred Hutchinson Cancer Research Center in Seattle, WA, and the Karolinska Institute, Stockholm, Sweden. According to these criteria, patients are classified into risk groups depending on steroid dose (immunosuppression), T-cell depletion, CD34 marker and days post-transplant. According to the risk group, the minimum clinical thresholds range from 100 viral copies to 1000 viral copies per ml of plasma or whole blood. Supplementary Figure S2 shows the HCMV DNA copies detected in our samples in the context of the clinical threshold.

We measured the viral loads in plasma and leukocytes to reflect viruses expanding in lytic or latent cycles. However, the source of the DNA observed in plasma may also come from the lysis of cells and not necessarily from lytic reactivation.

We were unable to collect all the EBV and HCMV serological data of the donor-receptor pairs (Supplementary Table S1) to address whether mismatches between them favor viral detection.

Finally, we do not have the serology results for the controls used in the study. However, in our own experience in working with EBV serology in the Mexican population, we have found that by age 30, 94% of the population is EBV-positive [73], and in two different pediatric cohorts, we found that 64.3% were positive by age 10 [74] and 69.8% by age 10 [75].

## Figures and Tables

**Table 1:** Patients (n = 40) and samples (n = 412).

Type of Transplant	Allogenic	Autologous
No. of patients	n = 32	n = 8
No. of blood samples	382	30
Samples per patient, mean ± SD	14 ± 6	4.6 ± 1.4
Follow-up, median days (IQR)	237 (88–338)	49 (23–80)
Sampling frequency, median days (IQR)	14 (14–24)	19 (13–28)
Sex, n (%)		
Female	16 (50)	5 (62.5)
Male	16 (50)	3 (37.5)
Age at transplant, median years (IQR)	8 (5–13)	10 (5–14.5)
Stem cell source, n (%)		
Peripheral blood	27 (84.4)	8 (100)
Bone marrow	5 (15.6)	0 (0)
HLA compatibility, n (%)		
50%	10 (31.3)	
75%	4 (12.5)	
80%	9 (28.1)	
100%	9 (28.1)	
Primary disease, n (%)		
Acute lymphoblastic leukemia	14 (43.8)	0 (0)
Acute myeloid leukemia	4 (12.5)	0 (0)
Aplastic anemia	5 (15.6)	0 (0)
Ewing sarcoma	0 (0)	4 (50)
Neuroblastoma	0 (0)	2 (25)
Other	9 (28.1)	2 (25)
Detection frequency of viral DNA (%)		
EBV	43.7	30
HCMV	5	6.7
HHV6	7.9	20
HHV7	9.7	23.3
Graft-versus-host disease, n (%)	25 (78)	
Mortality, n (%)	At 365 dayspost-transplant	At 100 dayspost-transplant
	13 (40.6)	3 (37.5)

## Supplementary Materials

The following supporting information can be downloaded at: https://www.mdpi.com/article/10.3390/microorganisms10081685/s1, Figure S1: Kinetics of infections in childhood allogeneic and autologous hematopoietic stem cell transplant (HSCT). The follow up is shown in the X axis and the viral load in the Y axis. Each virus is represented with a specific color. Viral loads are expressed as DNA copies/ug DNA in leukocytes or ml plasma; Figure S2: Viral loads for HCMV in patients with allogeneic and autologous hematopoietic stem cell transplantation (HSCT). Viral loads are expressed as DNA copies/ug of DNA in leukocytes or ml of plasma. The red line indicates the clinical threshold used at the Children’s Hospital of Mexico; Table S1: Pre-transplant serology for donors and recipients; Table S2: Leukocyte counts in blood samples.
