# Supplementary material for: Herpesvirus Screening in Childhood Hematopoietic Transplant Reveals High Systemic Inflammation in Episodes of Multiple Viral Detection and an EBV Association with Elevated IL-1β, IL-8 and Graft-Versus-Host Disease
Source: Microorganisms. 2022 Aug 22;10(8):1685. doi: 10.3390/microorganisms10081685 (PMC9414306; doi:10.3390/microorganisms10081685)
Supplement: Supplementary file 1 [file microorganisms-10-01685-s001.zip › microorganisms-1826016-supplementary.pdf]

Supplementary Figure S1. Kinetics of infections in childhood allogeneic and autologous hematopoietic stem cell transplant (HSCT). The follow up is shown in the X axis and the viral load in the Y axis. Each virus is represented with a specific color. Viral loads are expressed as DNA copies/ ug DNA in leukocytes or ml plasma.

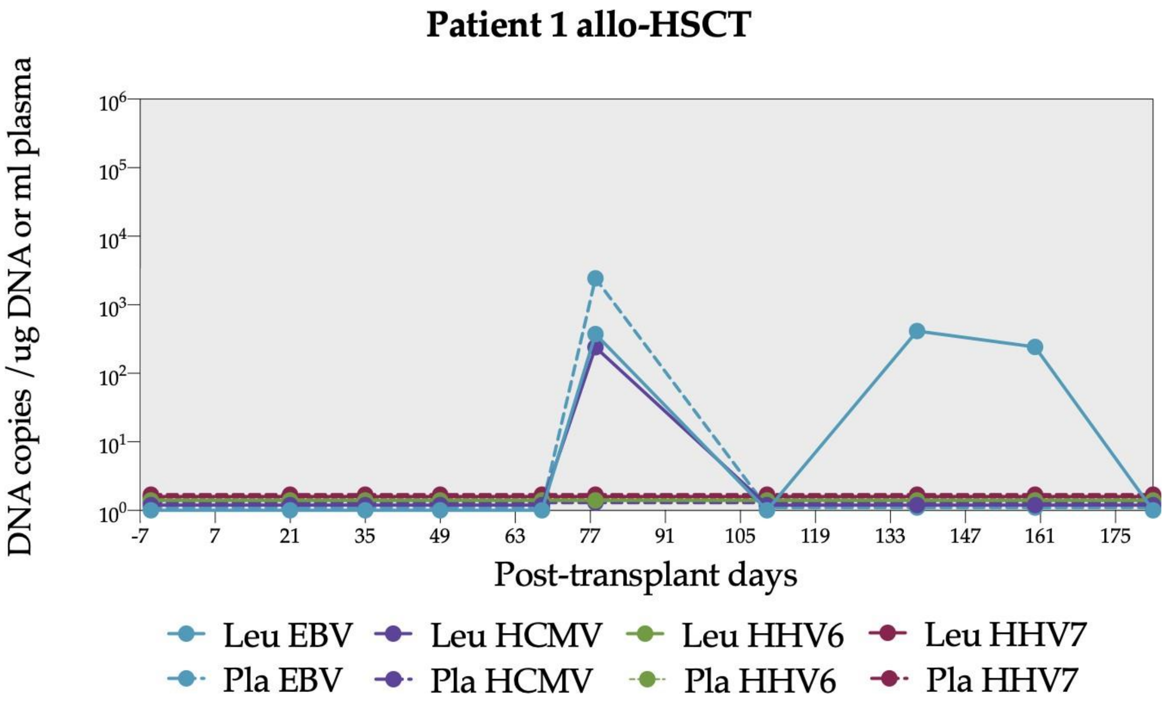

(a). Kinetics of infections in patient 1 who received allogeneic hematopoietic stem celltransplant (HSCT). The follow up is shown in the X axis and the viral load in the Y axis. Each virus is representedwith a specific color. Viral loads are expressed as DNA copies/ ug DNA in leukocytes or ml plasma.

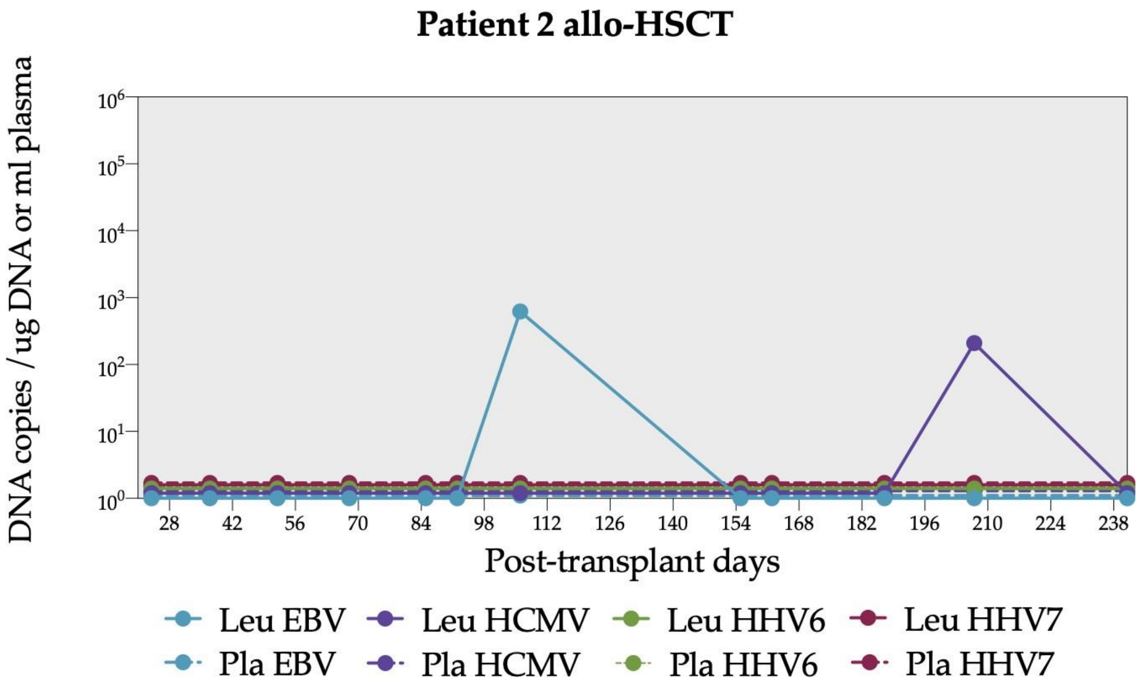

(b). Kinetics of infections in patient 2 who received allogeneic hematopoietic stem celltransplant (HSCT). The follow up is shown in the X axis and the viral load in the Y axis. Each virus is representedwith a specific color. Viral loads are expressed as DNA copies/ ug DNA in leukocytes or ml plasma.

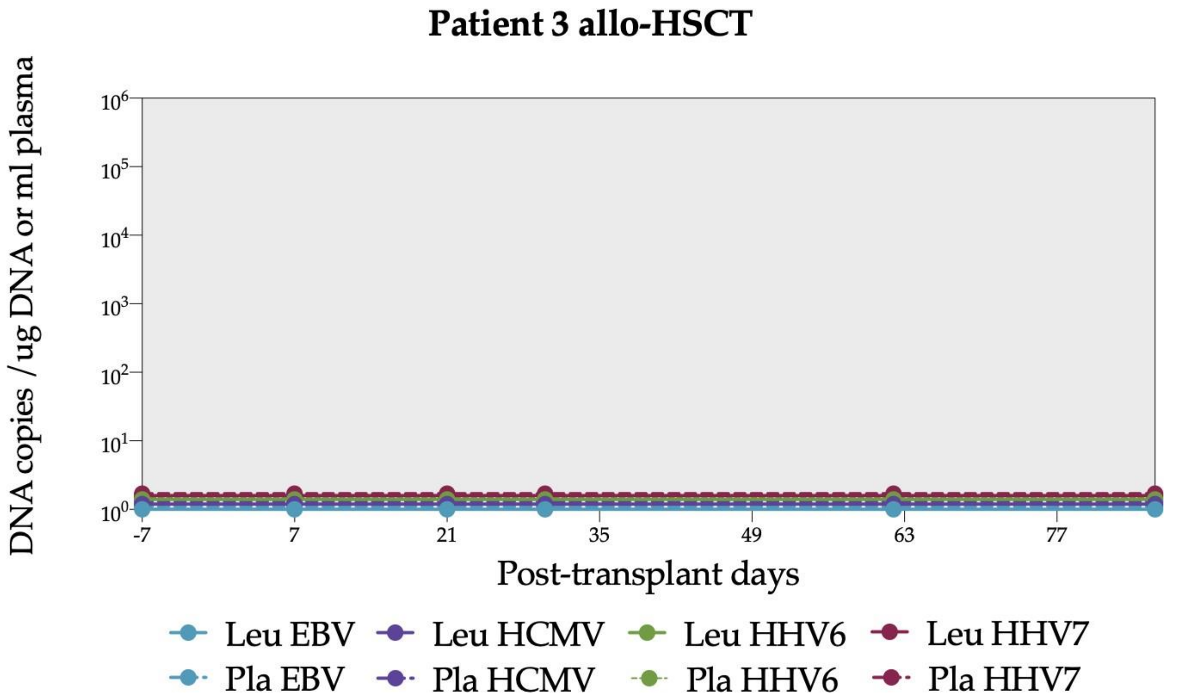

(c). Kinetics of infections in patient 3 who received allogeneic hematopoietic stem celltransplant (HSCT). The follow up is shown in the X axis and the viral load in the Y axis. Each virus is representedwith a specific color. Viral loads are expressed as DNA copies/ ug DNA in leukocytes or ml plasma.

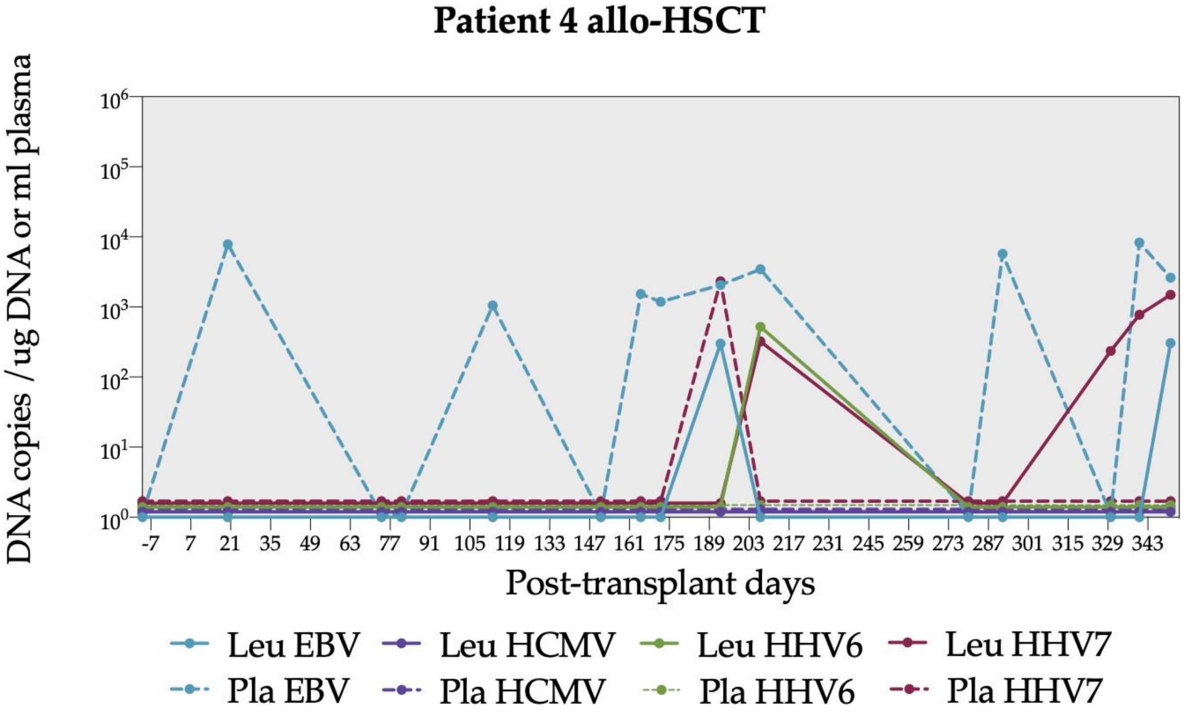

(d). Kinetics of infections in patient 4 who received allogeneic hematopoietic stem celltransplant (HSCT). The follow up is shown in the X axis and the viral load in the Y axis. Each virus is representedwith a specific color. Viral loads are expressed as DNA copies/ ug DNA in leukocytes or ml plasma.

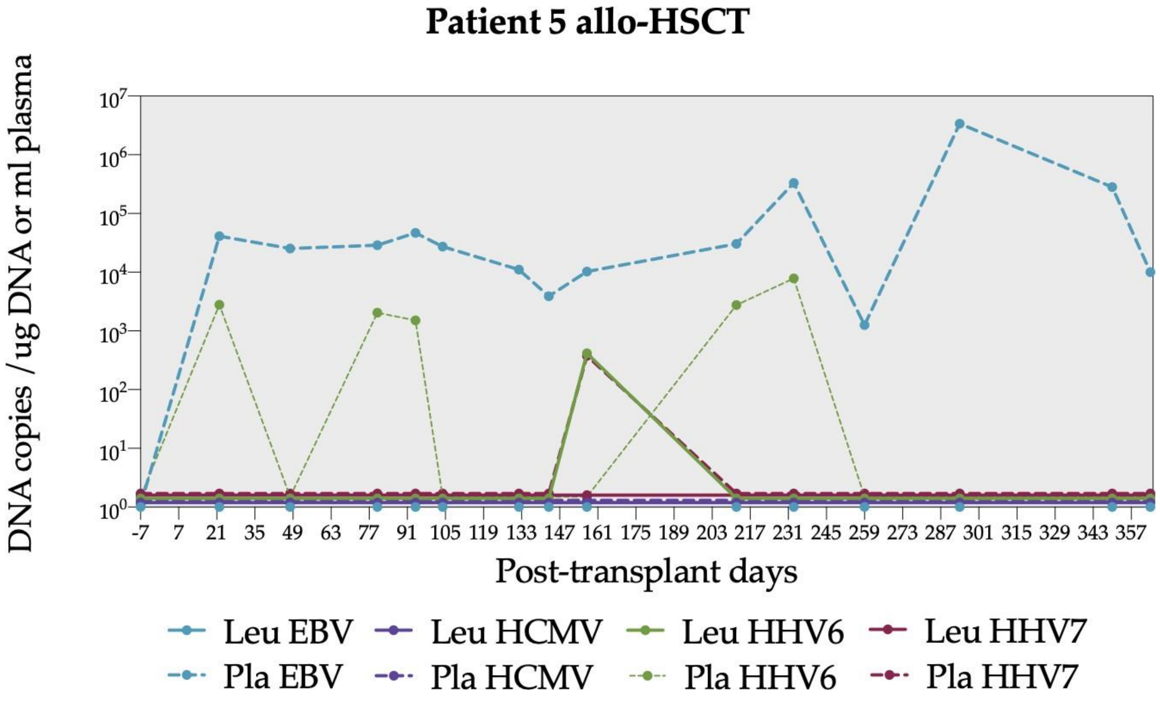

(e) Kinetics of infections in patient 5 who received allogeneic hematopoietic stem celltransplant (HSCT). The follow up is shown in the X axis and the viral load in the Y axis. Each virus is representedwith a specific color. Viral loads are expressed as DNA copies/ ug DNA in leukocytes or ml plasma.

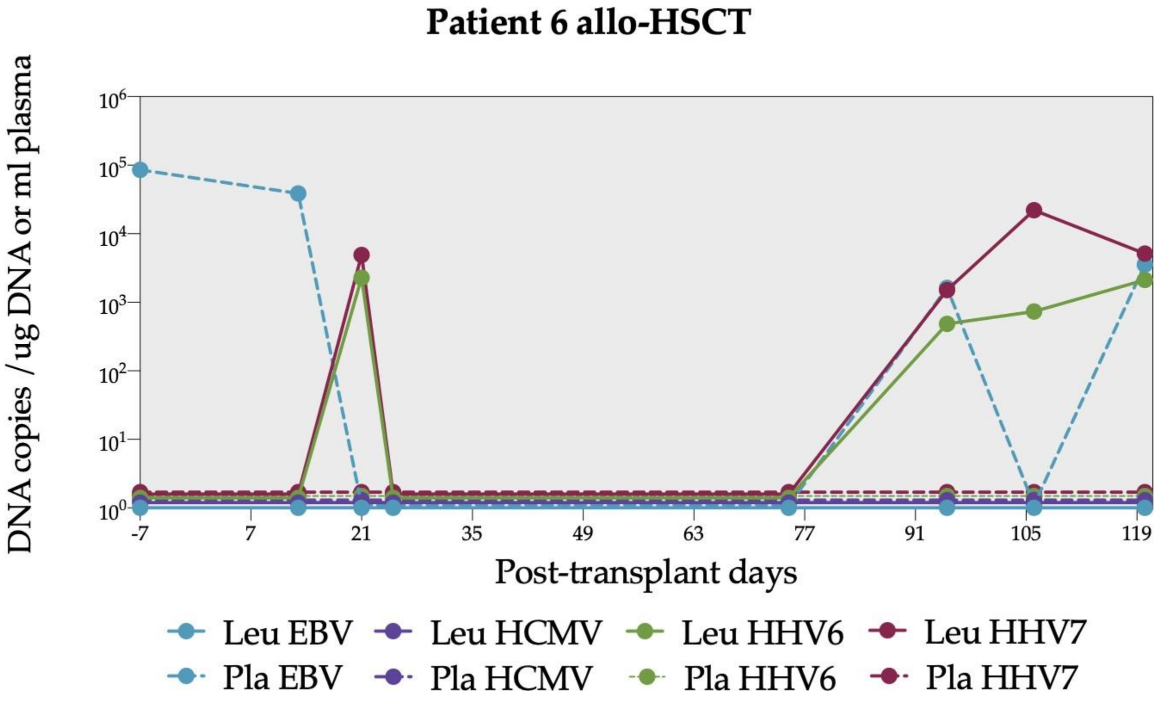

(f) Kinetics of infections in patient 6 who received allogeneic hematopoietic stem celltransplant (HSCT). The follow up is shown in the X axis and the viral load in the Y axis. Each virus is representedwith a specific color. Viral loads are expressed as DNA copies/ ug DNA in leukocytes or ml plasma.

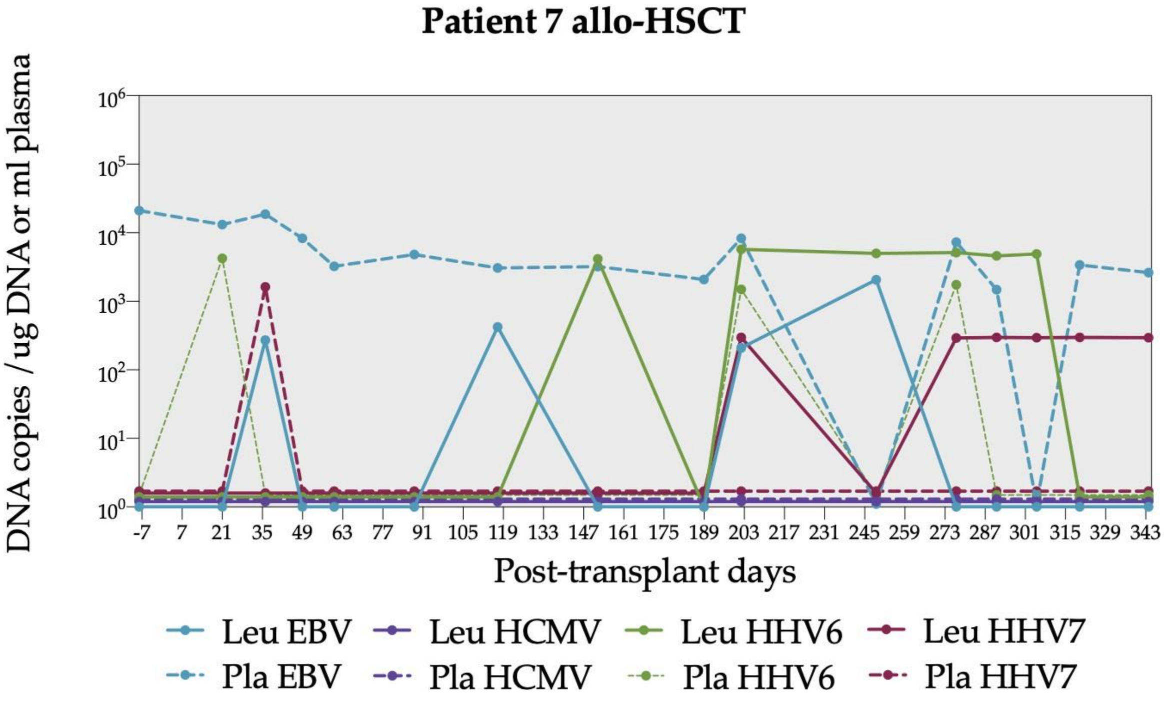

(g) Kinetics of infections in patient 7 who received allogeneic hematopoietic stem celltransplant (HSCT). The follow up is shown in the X axis and the viral load in the Y axis. Each virus is representedwith a specific color. Viral loads are expressed as DNA copies/ ug DNA in leukocytes or ml plasma.

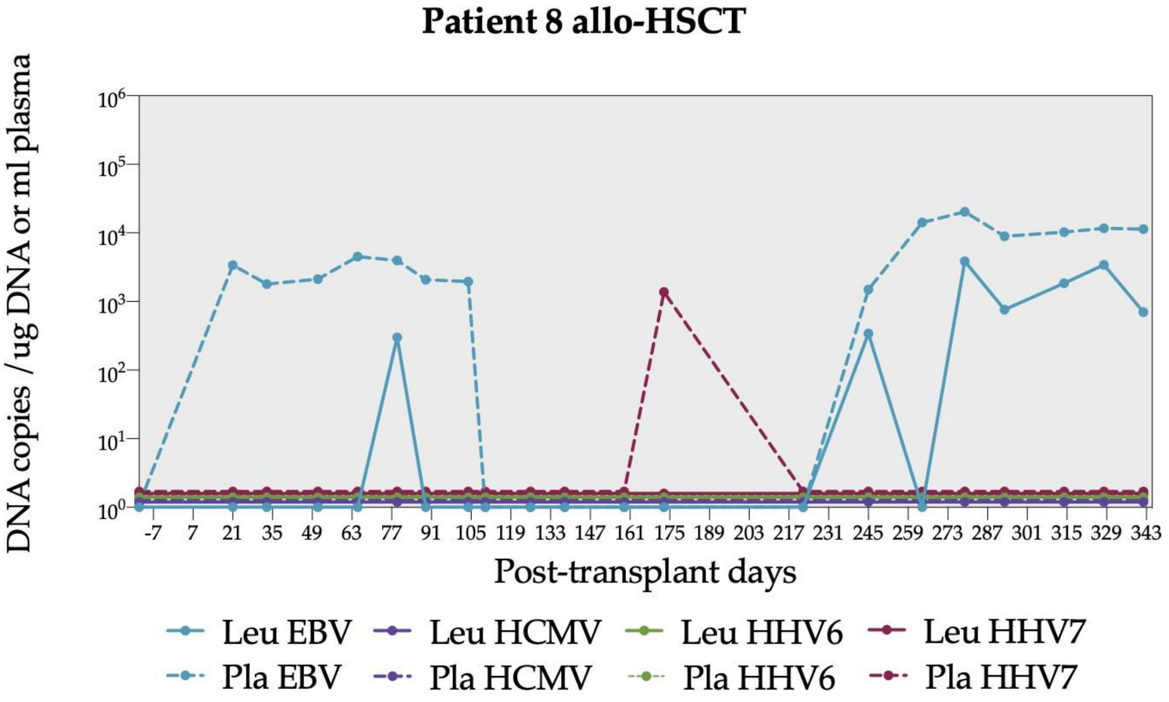

(h) Kinetics of infections in patient 8 who received allogeneic hematopoietic stem celltransplant (HSCT). The follow up is shown in the X axis and the viral load in the Y axis. Each virus is representedwith a specific color. Viral loads are expressed as DNA copies/ ug DNA in leukocytes or ml plasma.

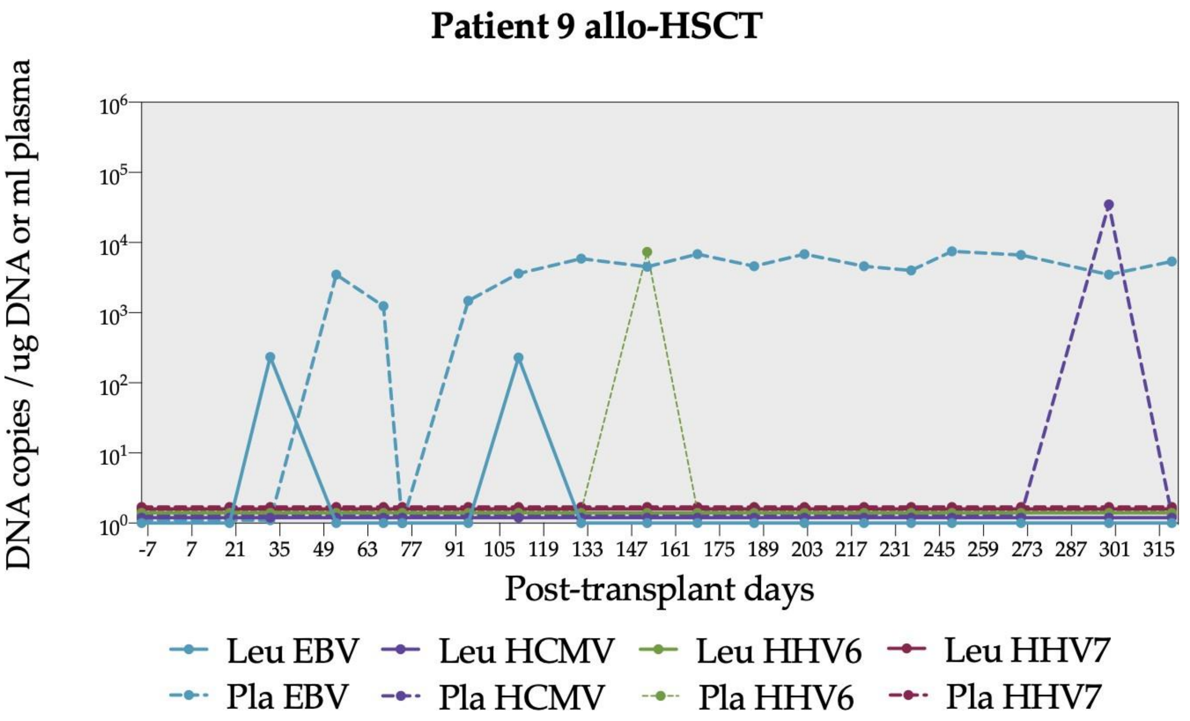

(i) Kinetics of infections in patient 9 who received allogeneic hematopoietic stem celltransplant (HSCT). The follow up is shown in the X axis and the viral load in the Y axis. Each virus is representedwith a specific color. Viral loads are expressed as DNA copies/ ug DNA in leukocytes or ml plasma.

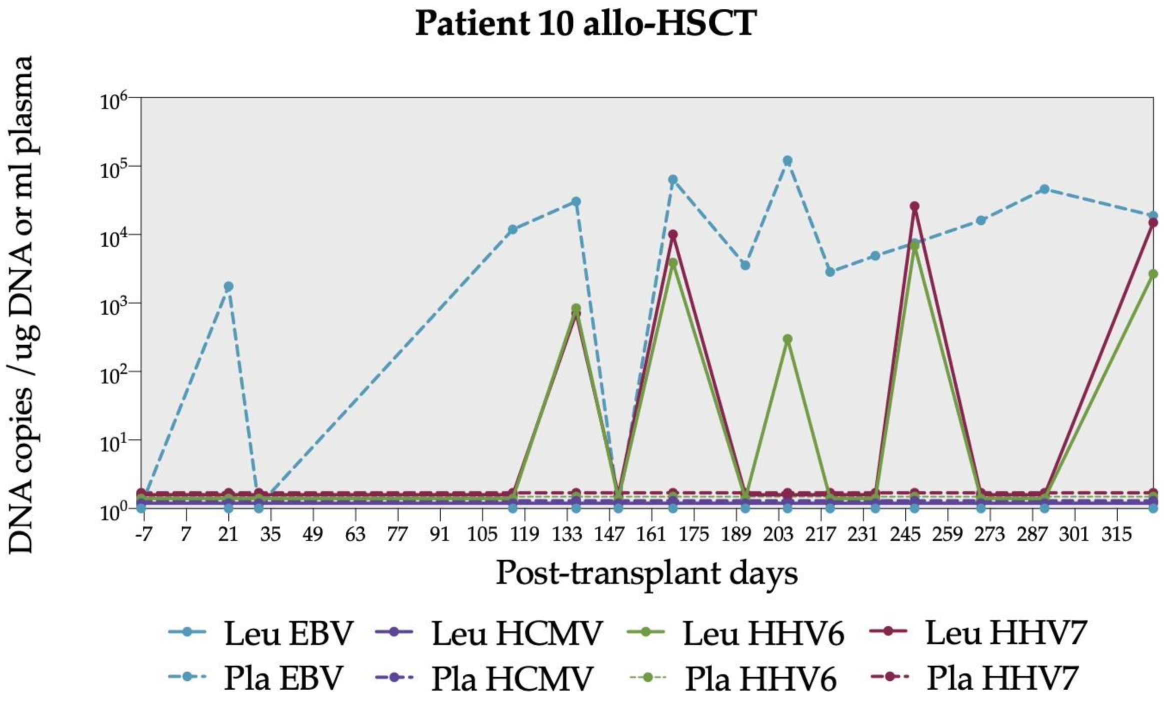

(j) Kinetics of infections in patient 10 who received allogeneic hematopoietic stem celltransplant (HSCT). The follow up is shown in the X axis and the viral load in the Y axis. Each virus is representedwith a specific color. Viral loads are expressed as DNA copies/ ug DNA in leukocytes or ml plasma.

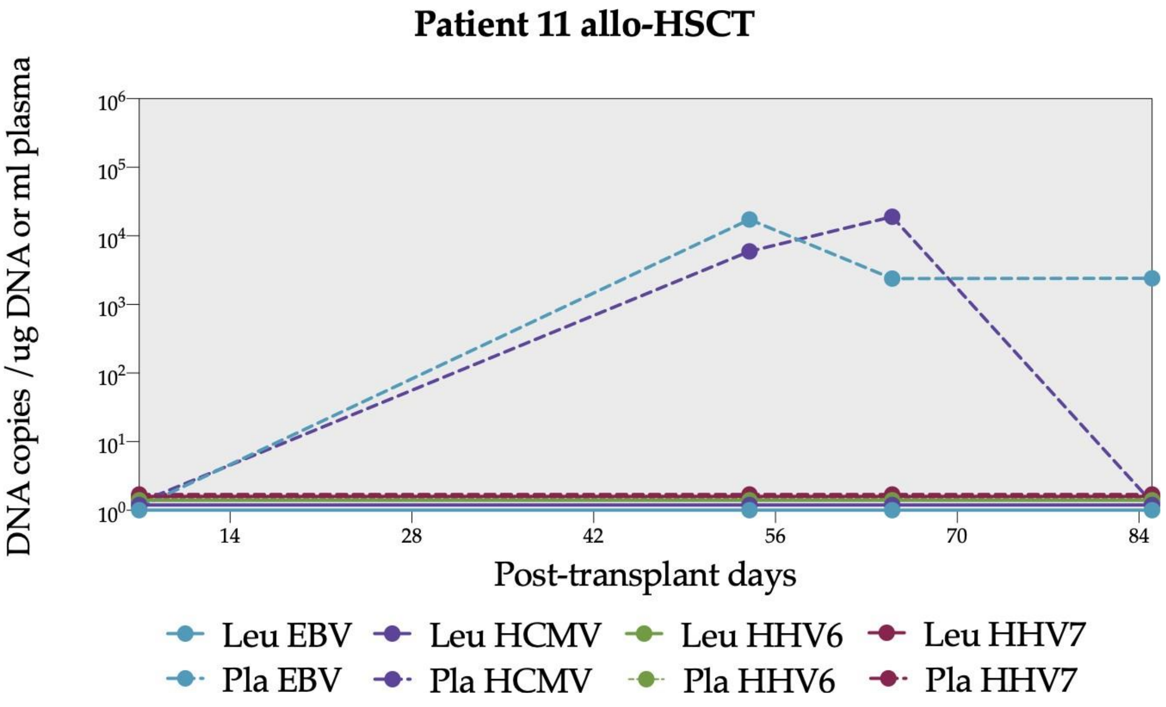

(k) Kinetics of infections in patient 11 who received allogeneic hematopoietic stem celltransplant (HSCT). The follow up is shown in the X axis and the viral load in the Y axis. Each virus is representedwith a specific color. Viral loads are expressed as DNA copies/ ug DNA in leukocytes or ml plasma.

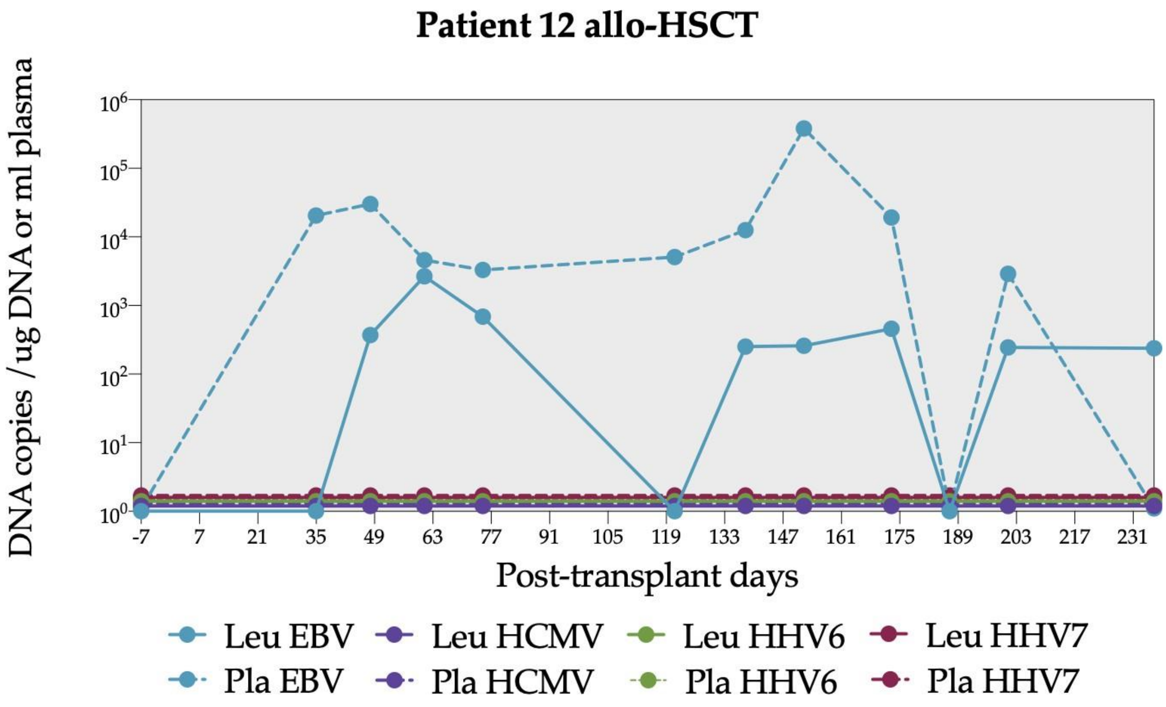

(l) Kinetics of infections in patient 12 who received allogeneic hematopoietic stem celltransplant (HSCT). The follow up is shown in the X axis and the viral load in the Y axis. Each virus is representedwith a specific color. Viral loads are expressed as DNA copies/ ug DNA in leukocytes or ml plasma.

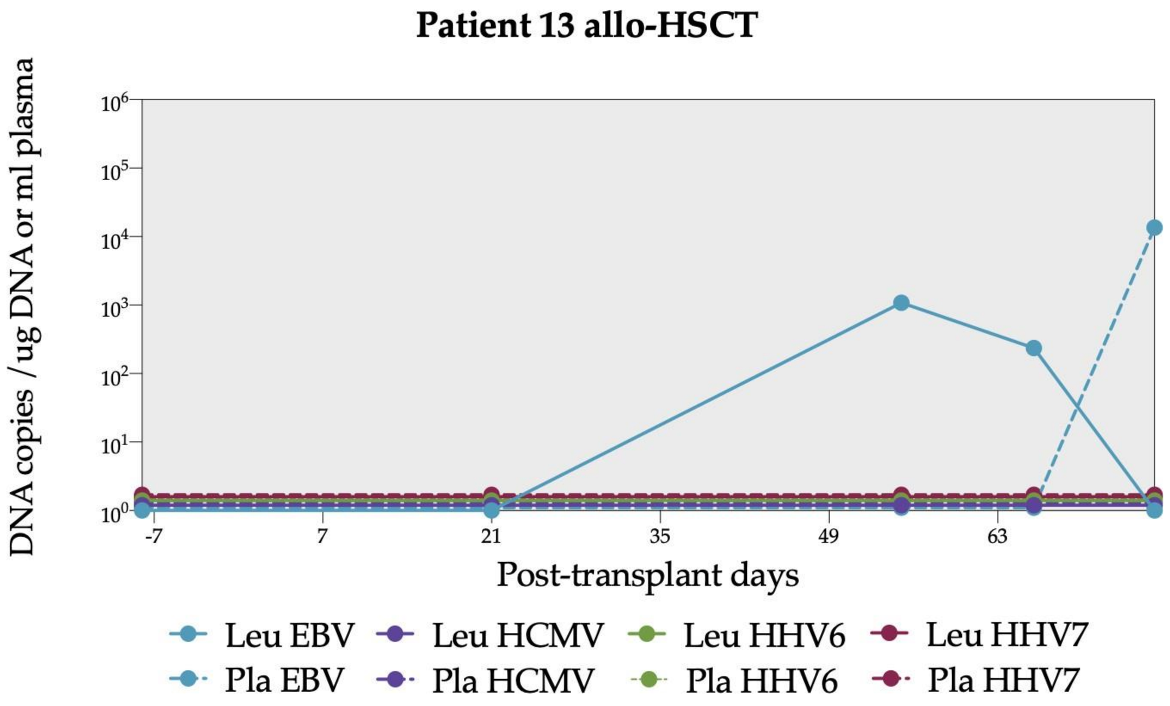

(m) Kinetics of infections in patient 13 who received allogeneic hematopoietic stem celltransplant (HSCT). The follow up is shown in the X axis and the viral load in the Y axis. Each virus is representedwith a specific color. Viral loads are expressed as DNA copies/ ug DNA in leukocytes or ml plasma.

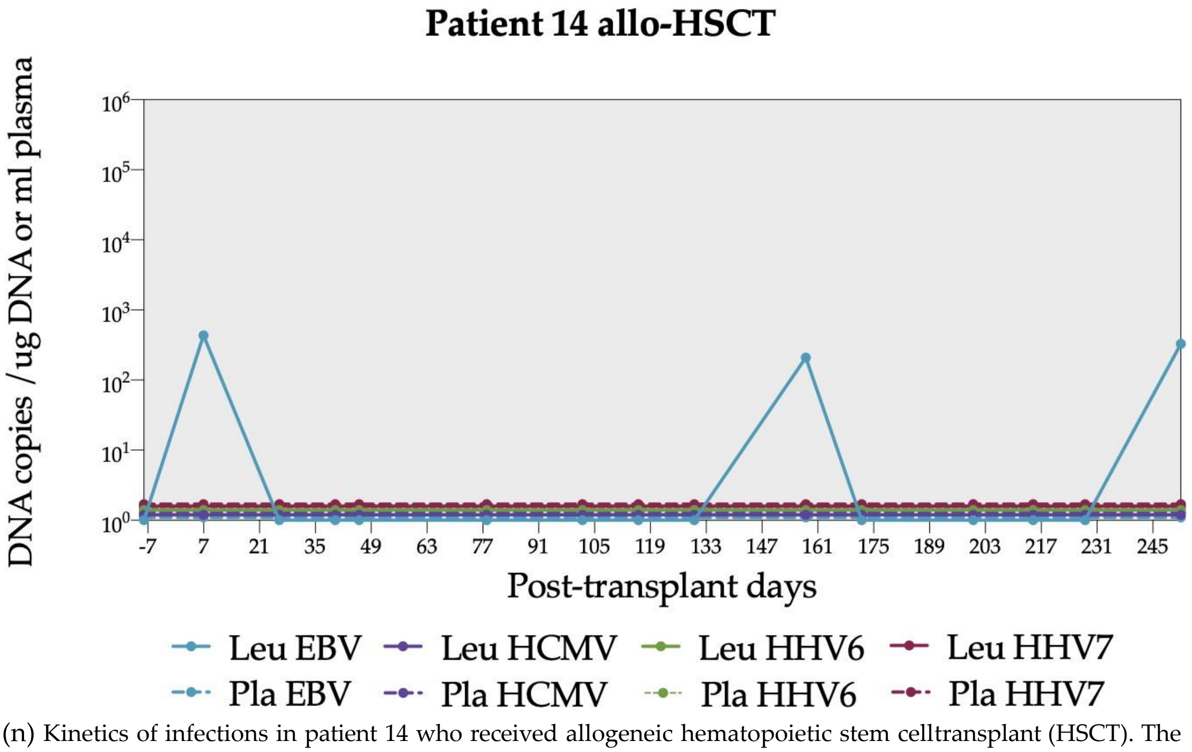

(n) Kinetics of infections in patient 14 who received allogeneic hematopoietic stem celltransplant (HSCT). The follow up is shown in the X axis and the viral load in the Y axis. Each virus is representedwith a specific color. Viral loads are expressed as DNA copies/ ug DNA in leukocytes or ml plasma.

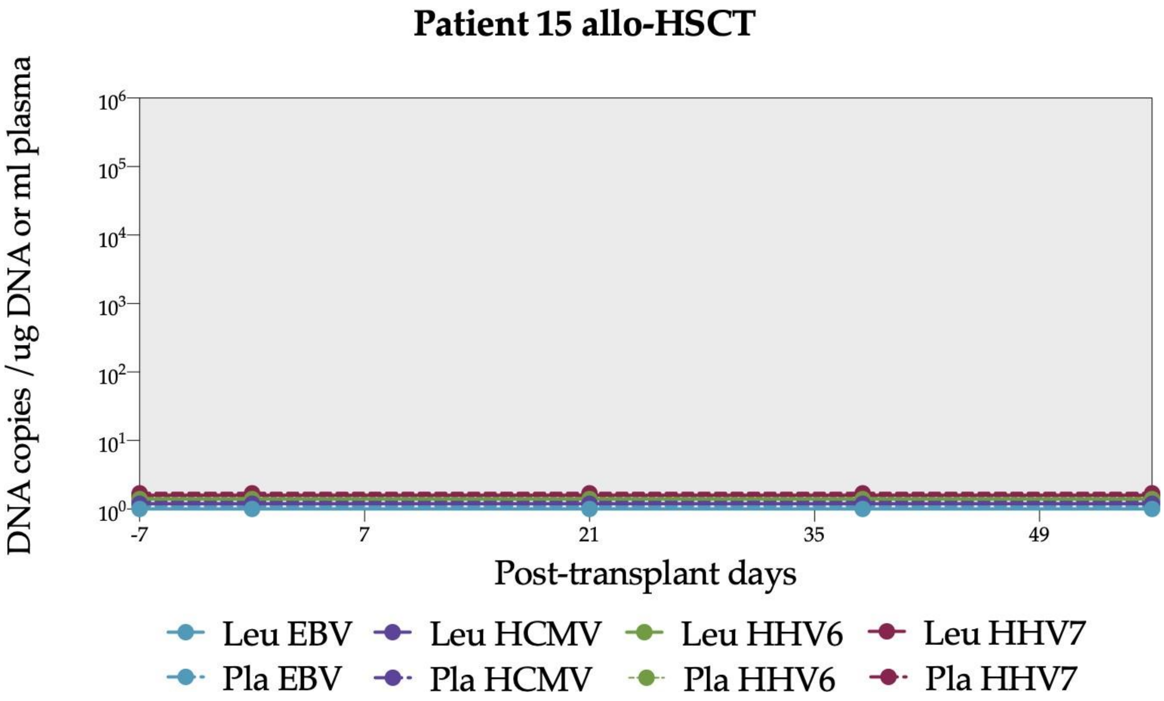

(o) Kinetics of infections in patient 15 who received allogeneic hematopoietic stem celltransplant (HSCT). The follow up is shown in the X axis and the viral load in the Y axis. Each virus is representedwith a specific color. Viral loads are expressed as DNA copies/ ug DNA in leukocytes or ml plasma.

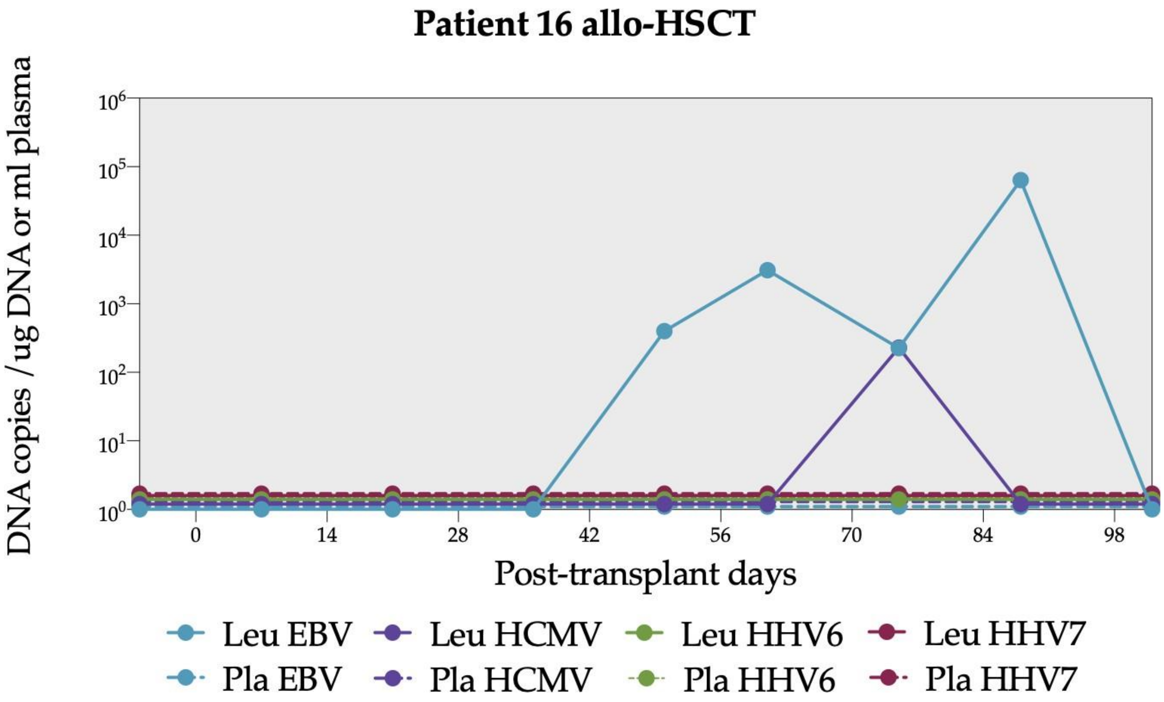

(p) Kinetics of infections in patient 16 who received allogeneic hematopoietic stem celltransplant (HSCT). The follow up is shown in the X axis and the viral load in the Y axis. Each virus is representedwith a specific color. Viral loads are expressed as DNA copies/ ug DNA in leukocytes or ml plasma.

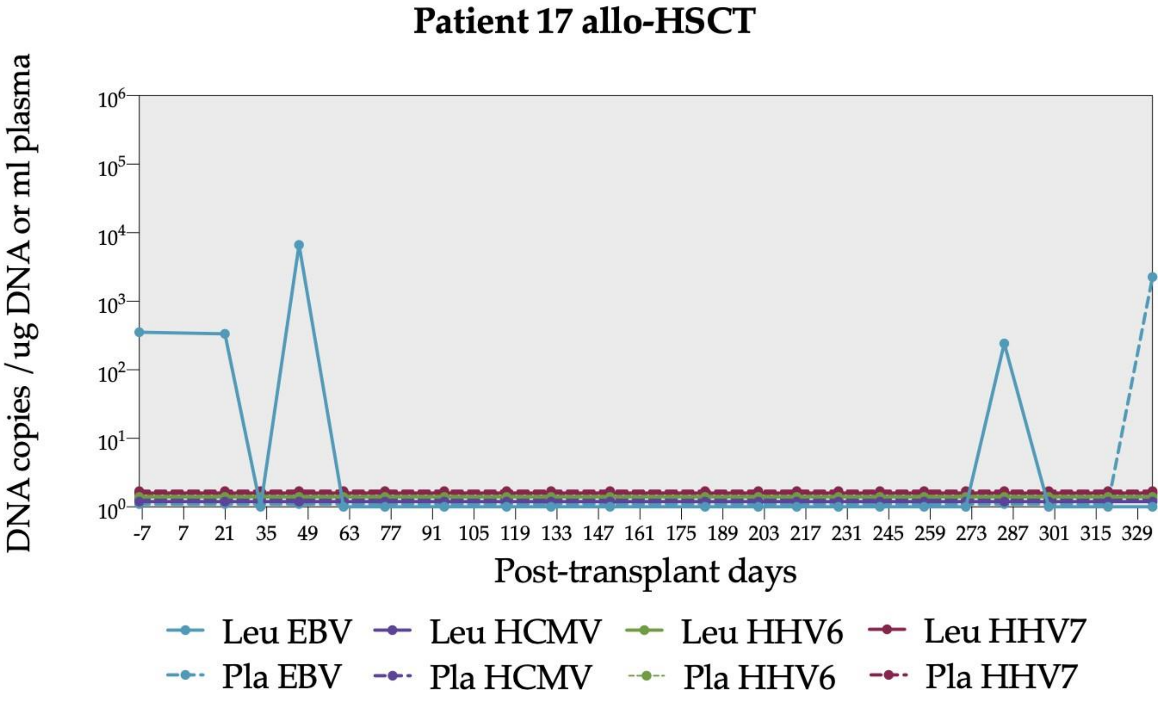

(q) Kinetics of infections in patient 17 who received allogeneic hematopoietic stem celltransplant (HSCT). The follow up is shown in the X axis and the viral load in the Y axis. Each virus is representedwith a specific color. Viral loads are expressed as DNA copies/ ug DNA in leukocytes or ml plasma.

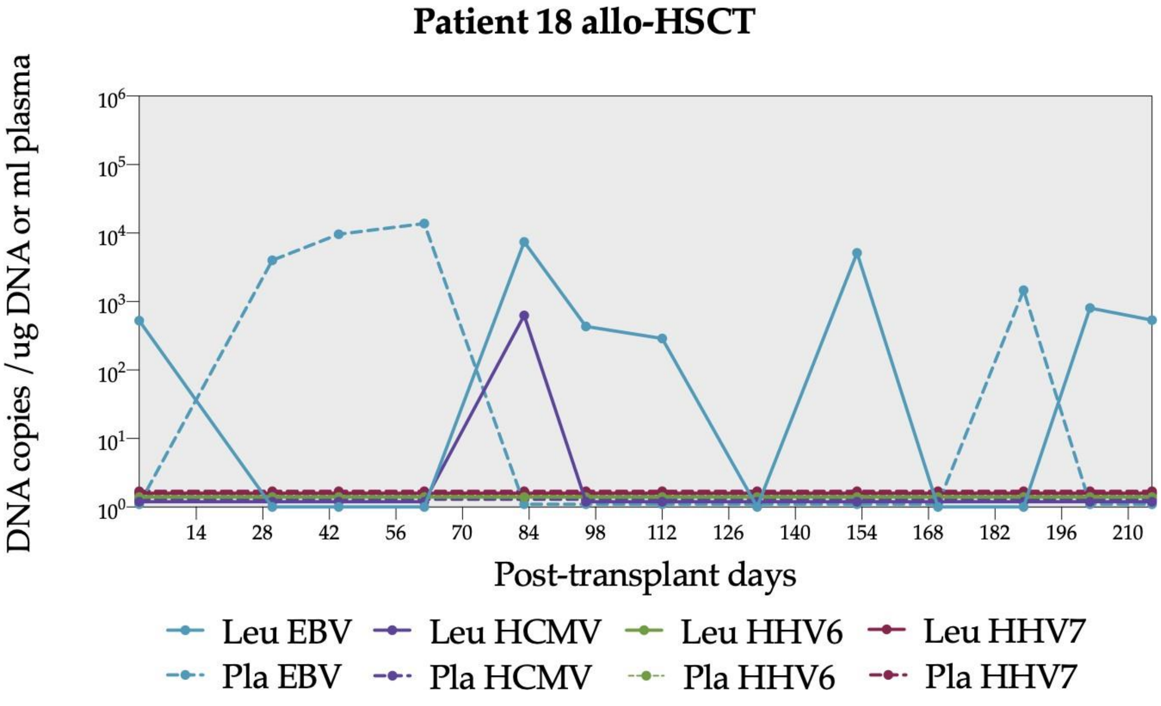

(r) Kinetics of infections in patient 18 who received allogeneic hematopoietic stem celltransplant (HSCT). The follow up is shown in the X axis and the viral load in the Y axis. Each virus is representedwith a specific color. Viral loads are expressed as DNA copies/ ug DNA in leukocytes or ml plasma.

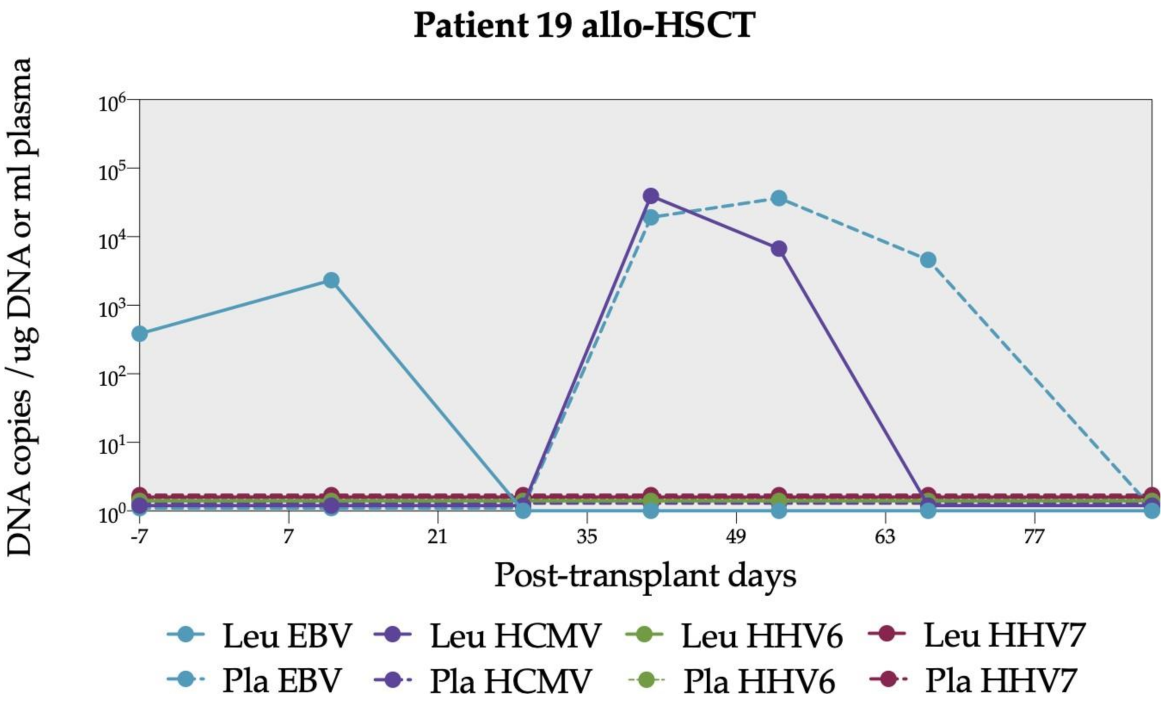

(s) Kinetics of infections in patient 19 who received allogeneic hematopoietic stem celltransplant (HSCT). The follow up is shown in the X axis and the viral load in the Y axis. Each virus is representedwith a specific color. Viral loads are expressed as DNA copies/ ug DNA in leukocytes or ml plasma.

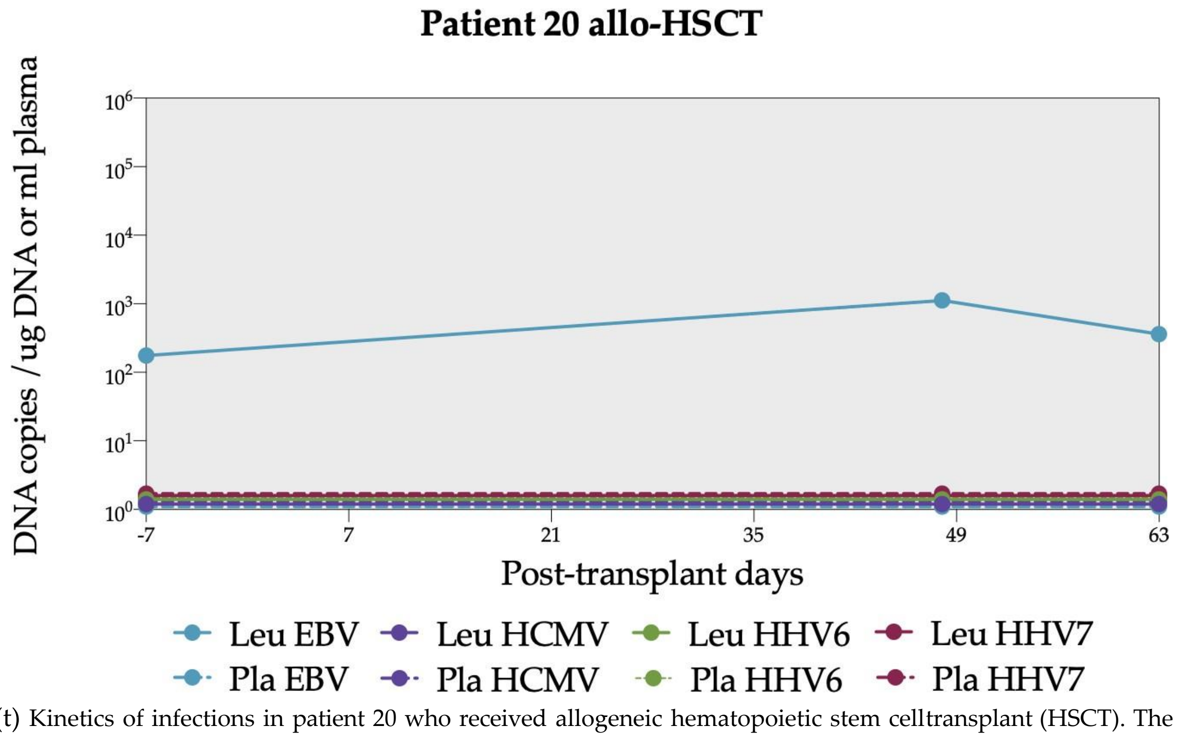

(t) Kinetics of infections in patient 20 who received allogeneic hematopoietic stem celltransplant (HSCT). The follow up is shown in the X axis and the viral load in the Y axis. Each virus is representedwith a specific color. Viral loads are expressed as DNA copies/ ug DNA in leukocytes or ml plasma.

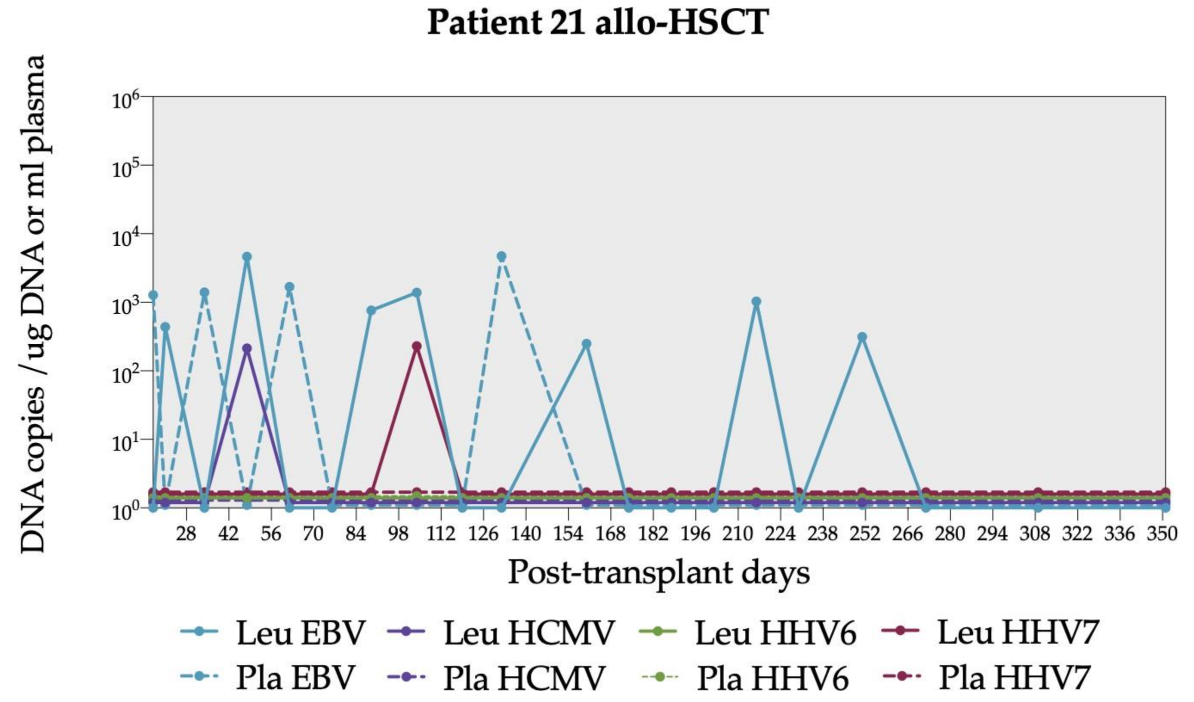

(u) Kinetics of infections in patient 21 who received allogeneic hematopoietic stem celltransplant (HSCT). The follow up is shown in the X axis and the viral load in the Y axis. Each virus is representedwith a specific color. Viral loads are expressed as DNA copies/ ug DNA in leukocytes or ml plasma.

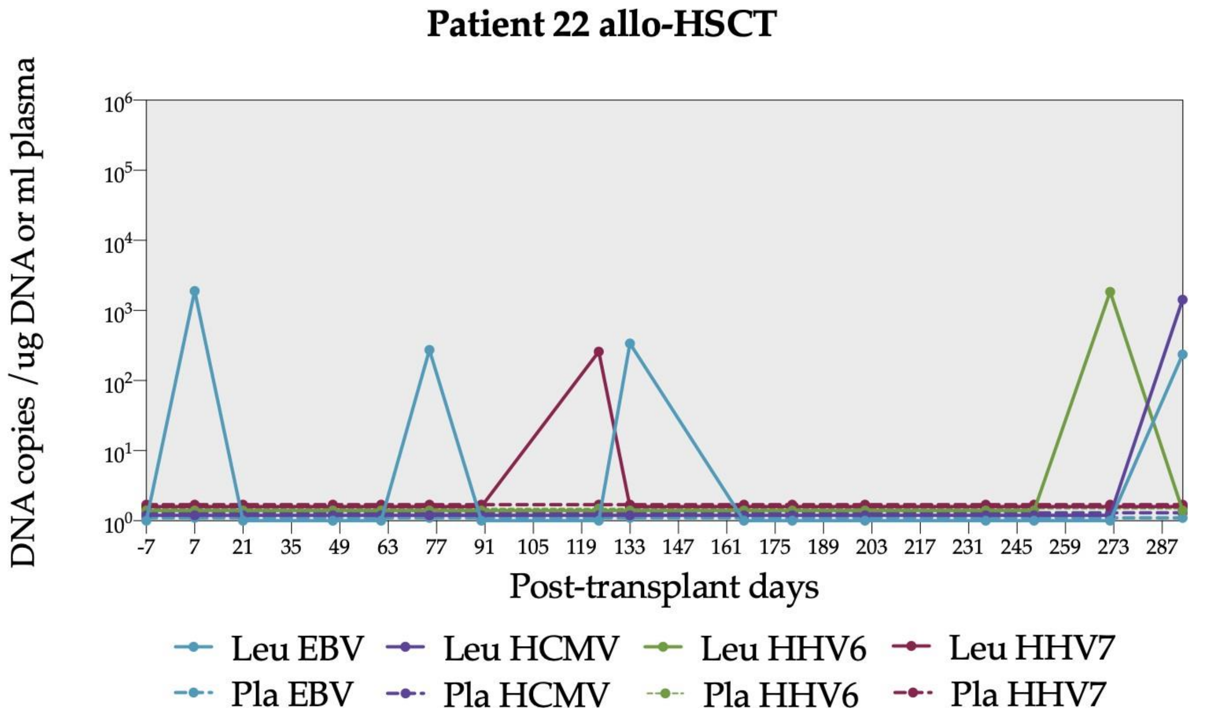

(v) Kinetics of infections in patient 22 who received allogeneic hematopoietic stem celltransplant (HSCT). The follow up is shown in the X axis and the viral load in the Y axis. Each virus is representedwith a specific color. Viral loads are expressed as DNA copies/ ug DNA in leukocytes or ml plasma.

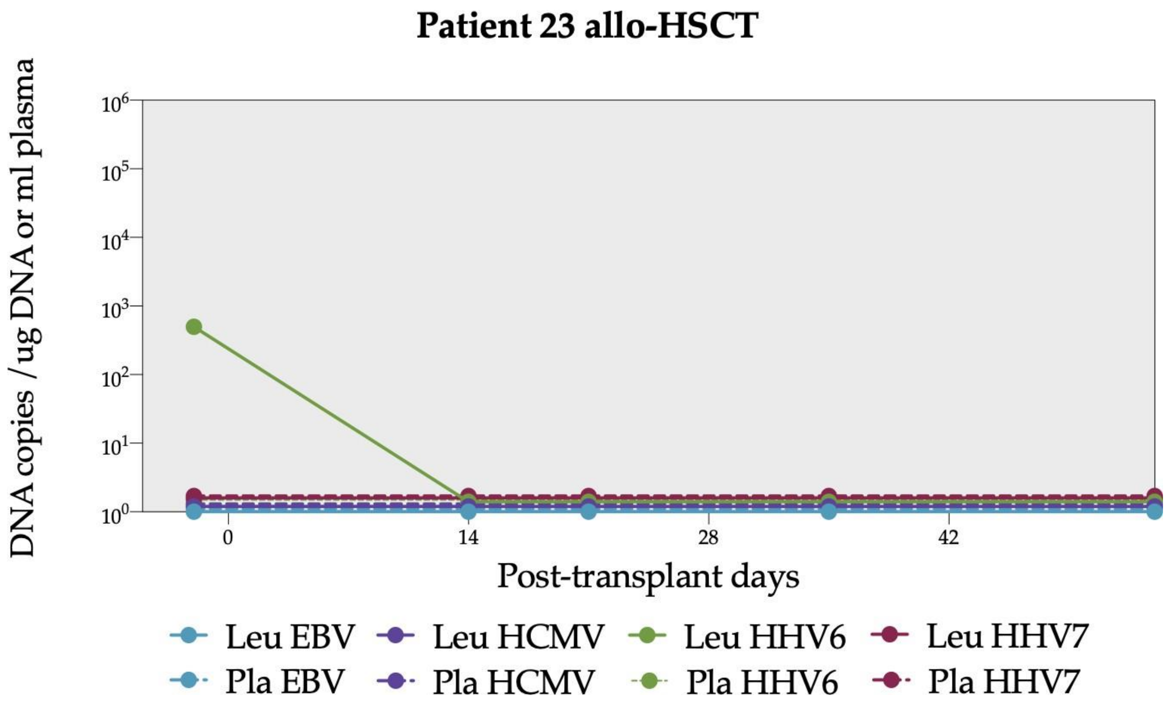

(w) Kinetics of infections in patient 23 who received allogeneic hematopoietic stem celltransplant (HSCT). The follow up is shown in the X axis and the viral load in the Y axis. Each virus is representedwith a specific color. Viral loads are expressed as DNA copies/ ug DNA in leukocytes or ml plasma.

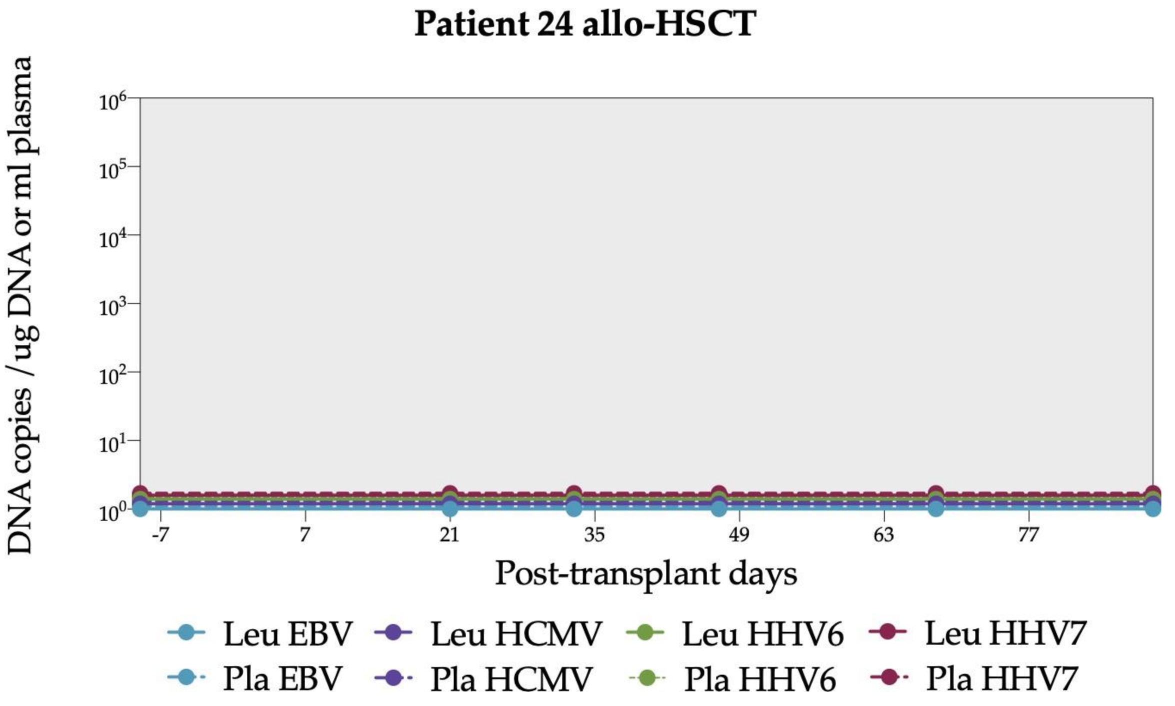

(x) Kinetics of infections in patient 24 who received allogeneic hematopoietic stem celltransplant (HSCT). The follow up is shown in the X axis and the viral load in the Y axis. Each virus is representedwith a specific color. Viral loads are expressed as DNA copies/ ug DNA in leukocytes or ml plasma.

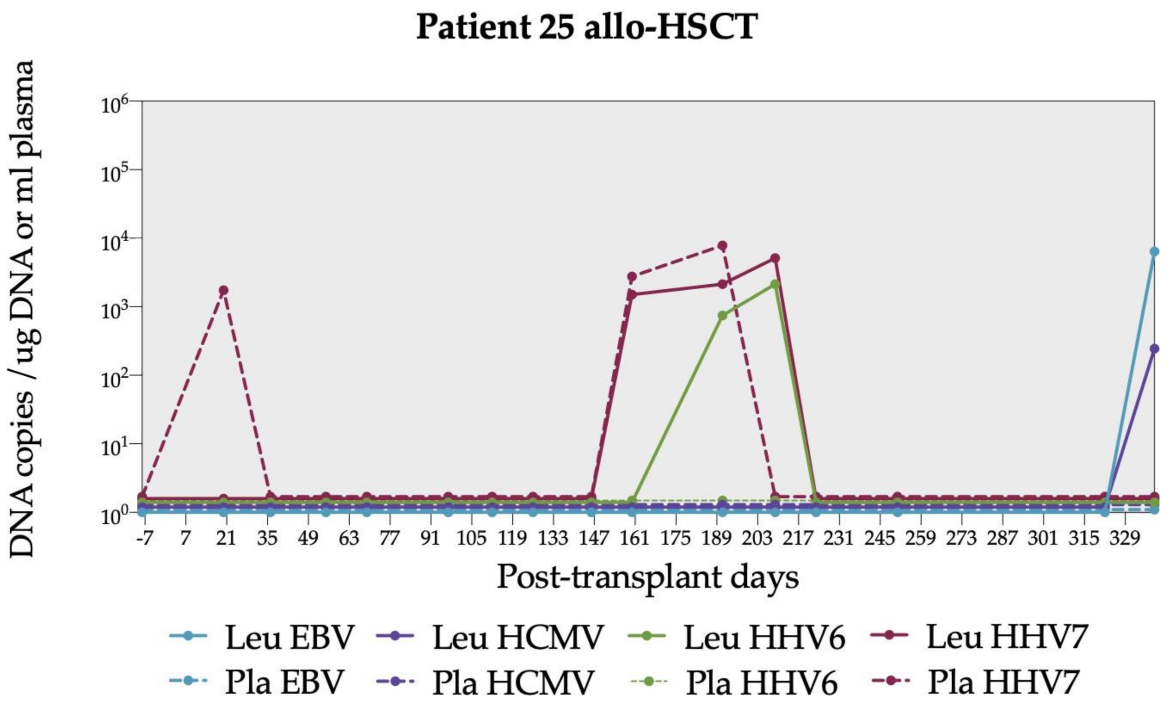

(y) Kinetics of infections in patient 25 who received allogeneic hematopoietic stem celltransplant (HSCT). The follow up is shown in the X axis and the viral load in the Y axis. Each virus is representedwith a specific color. Viral loads are expressed as DNA copies/ ug DNA in leukocytes or ml plasma.

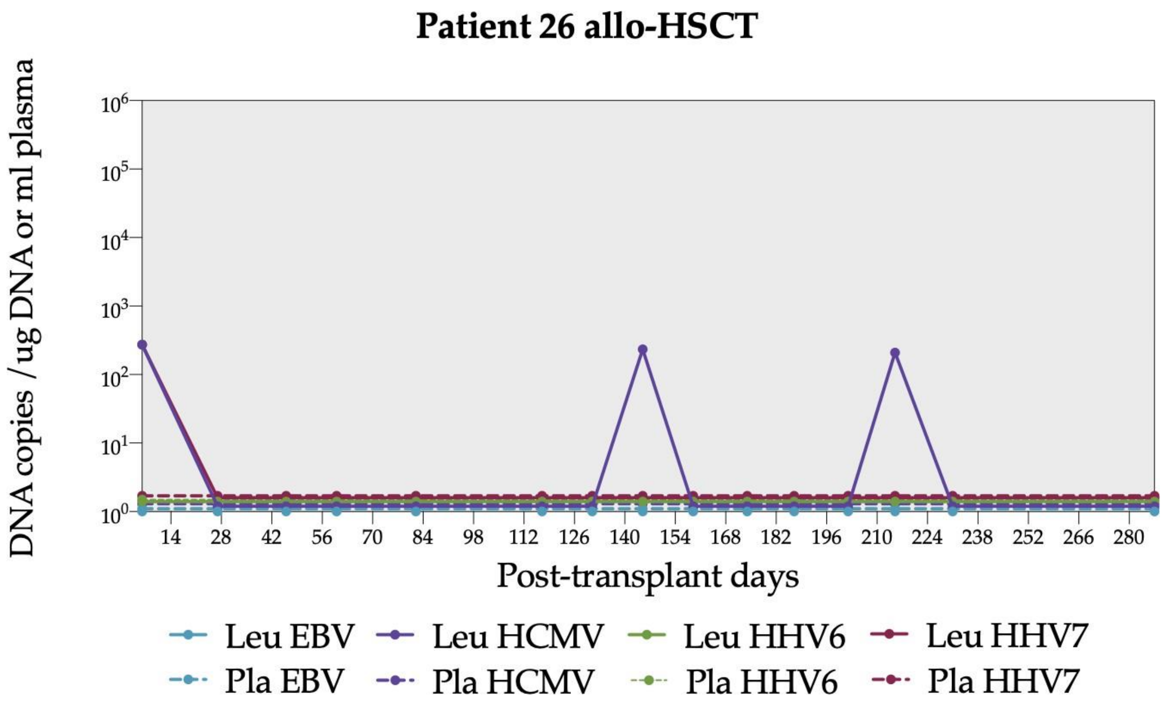

(z) Kinetics of infections in patient 26 who received allogeneic hematopoietic stem celltransplant (HSCT). The follow up is shown in the X axis and the viral load in the Y axis. Each virus is representedwith a specific color. Viral loads are expressed as DNA copies/ ug DNA in leukocytes or ml plasma.

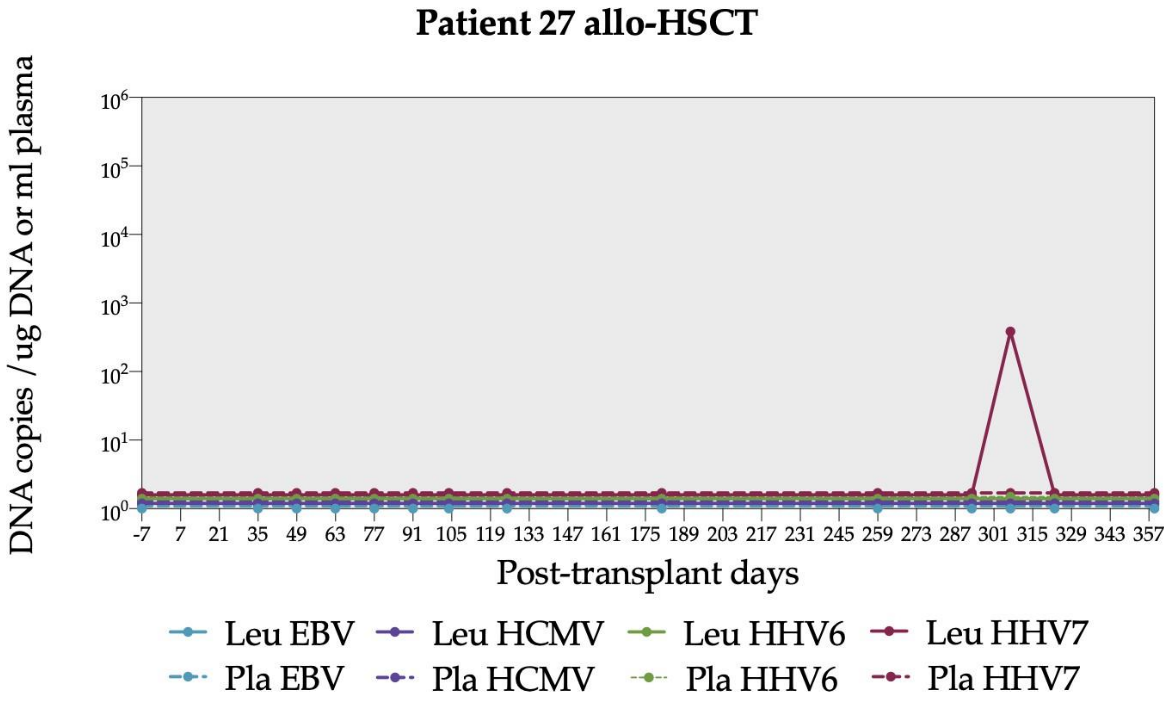

(aa) Kinetics of infections in patient 27 who received allogeneic hematopoietic stem celltransplant (HSCT). The follow up is shown in the X axis and the viral load in the Y axis. Each virus is representedwith a specific color. Viral loads are expressed as DNA copies/ ug DNA in leukocytes or ml plasma.

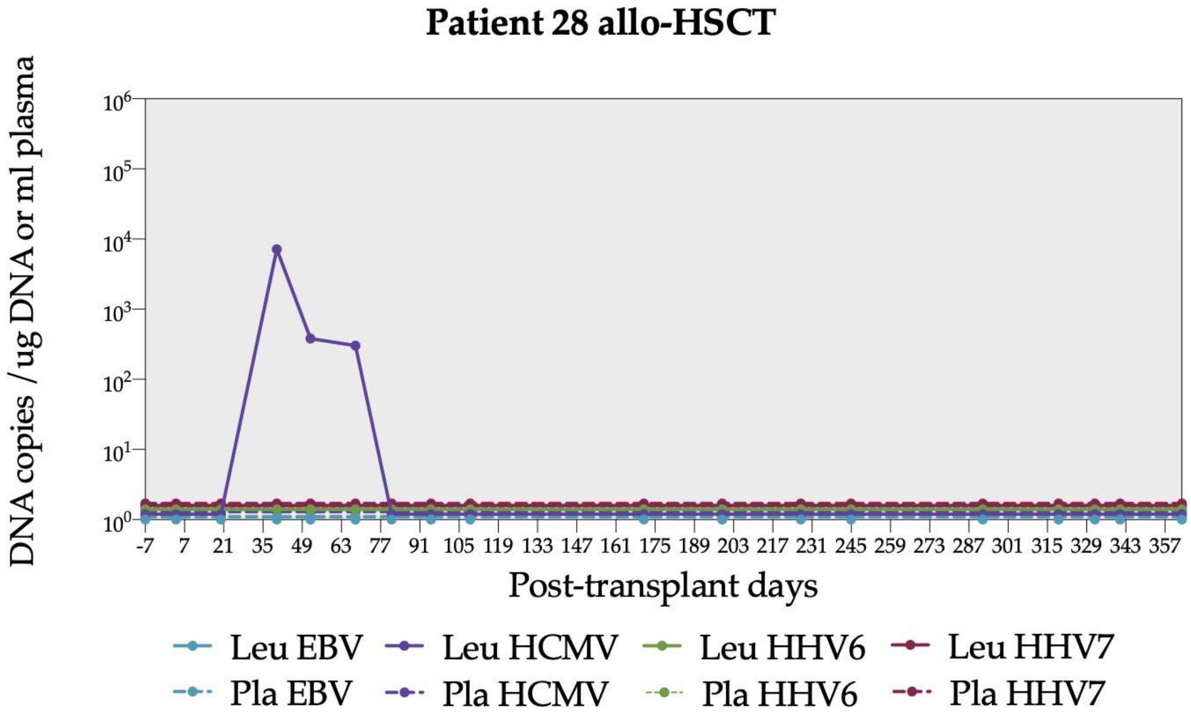

(ab) Kinetics of infections in patient 28 who received allogeneic hematopoietic stem celltransplant (HSCT). The follow up is shown in the X axis and the viral load in the Y axis. Each virus is representedwith a specific color. Viral loads are expressed as DNA copies/ ug DNA in leukocytes or ml plasma.

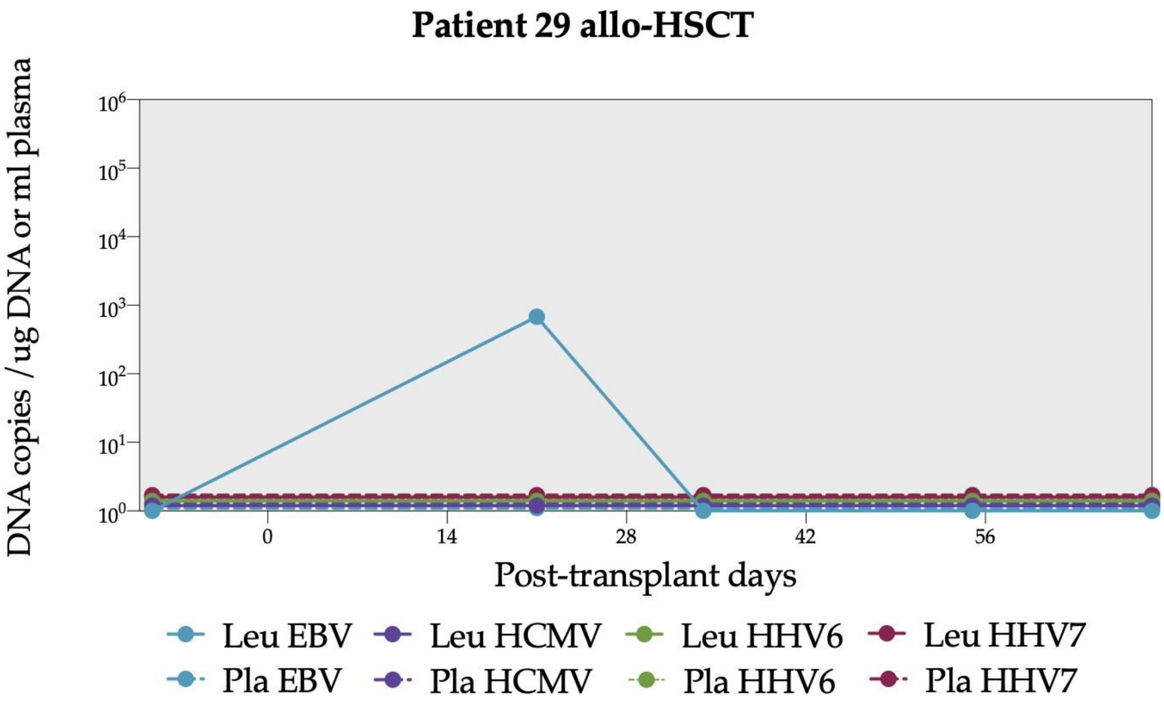

(ab) Kinetics of infections in patient 29 who received allogeneic hematopoietic stem celltransplant (HSCT). The follow up is shown in the X axis and the viral load in the Y axis. Each virus is representedwith a specific color. Viral loads are expressed as DNA copies/ ug DNA in leukocytes or ml plasma.

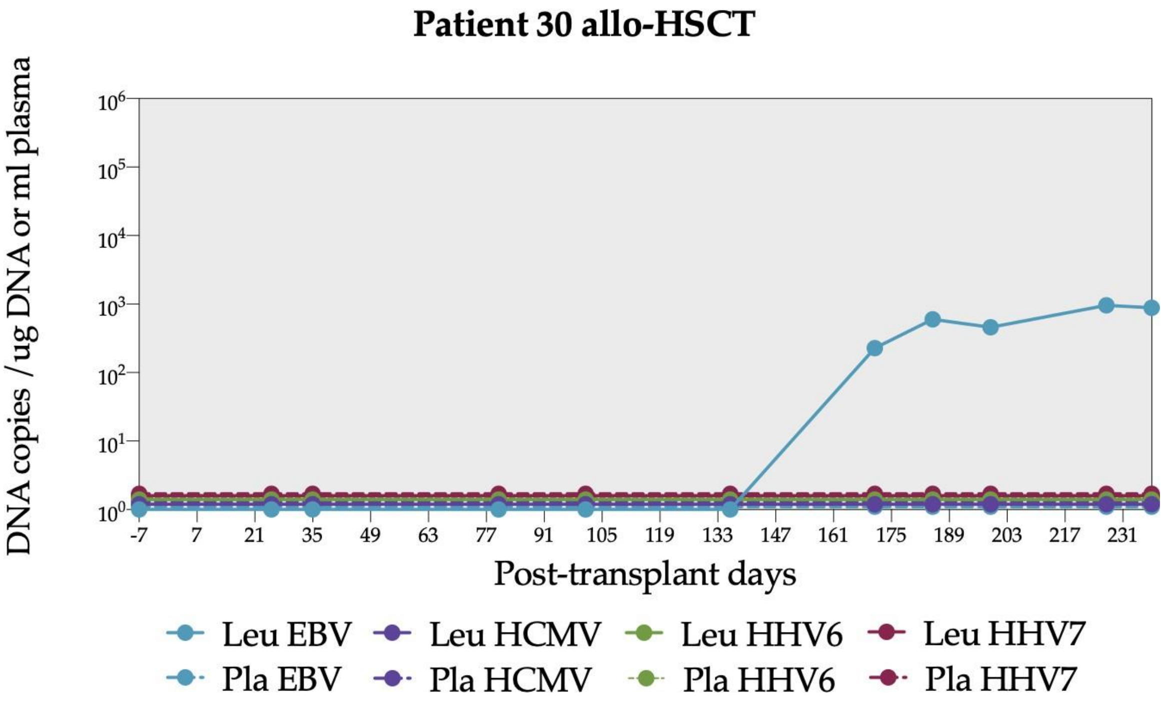

(ad) Kinetics of infections in patient 30 who received allogeneic hematopoietic stem celltransplant (HSCT). The follow up is shown in the X axis and the viral load in the Y axis. Each virus is representedwith a specific color. Viral loads are expressed as DNA copies/ ug DNA in leukocytes or ml plasma.

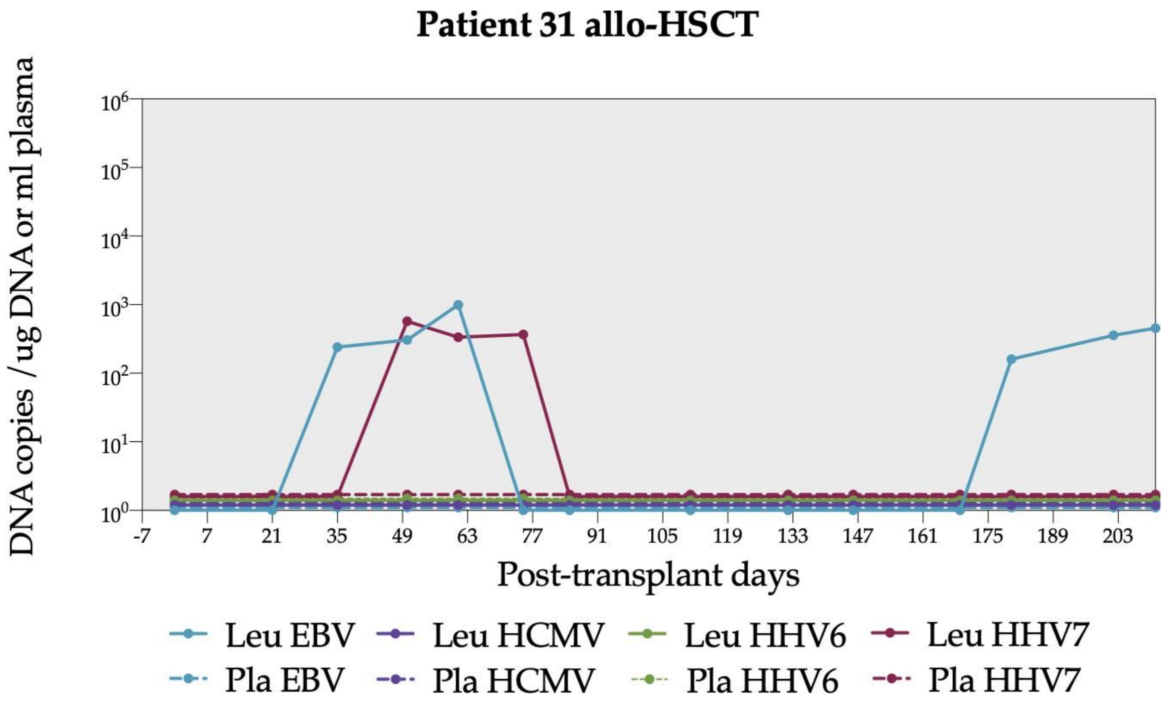

(ae) Kinetics of infections in patient 31 who received allogeneic hematopoietic stem celltransplant (HSCT). The follow up is shown in the X axis and the viral load in the Y axis. Each virus is representedwith a specific color. Viral loads are expressed as DNA copies/ ug DNA in leukocytes or ml plasma.

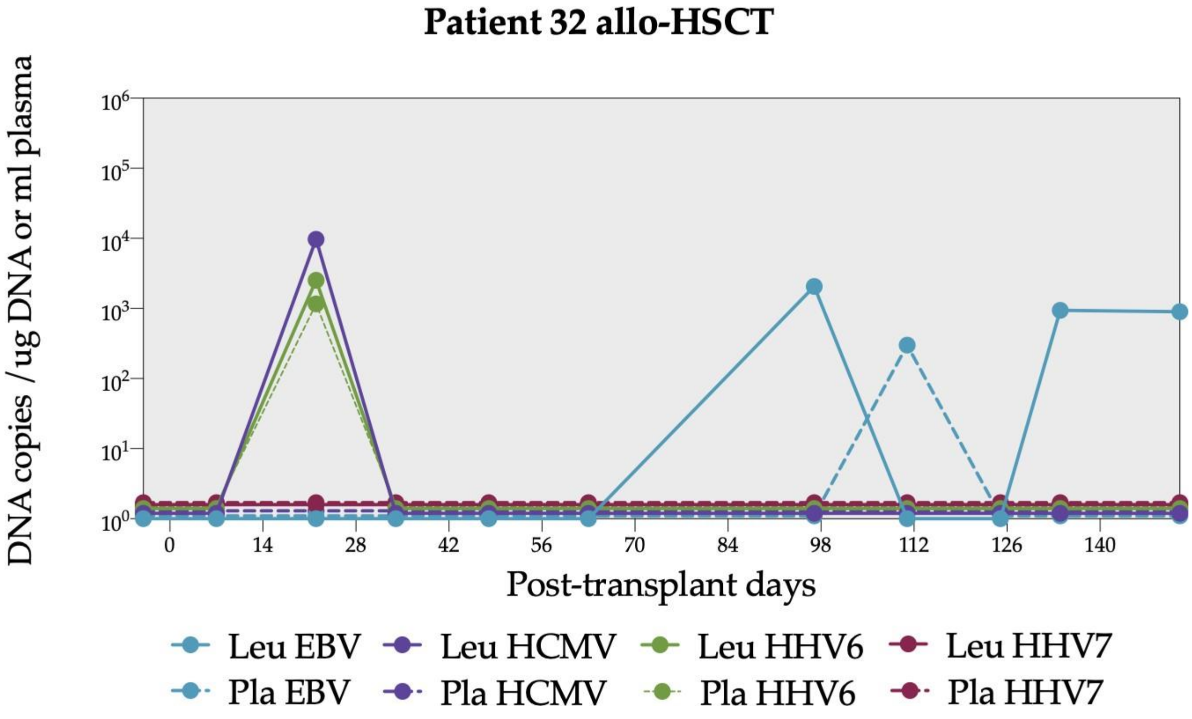

(af) Kinetics of infections in patient 32 who received allogeneic hematopoietic stem celltransplant (HSCT). The follow up is shown in the X axis and the viral load in the Y axis. Each virus is representedwith a specific color. Viral loads are expressed as DNA copies/ ug DNA in leukocytes or ml plasma.

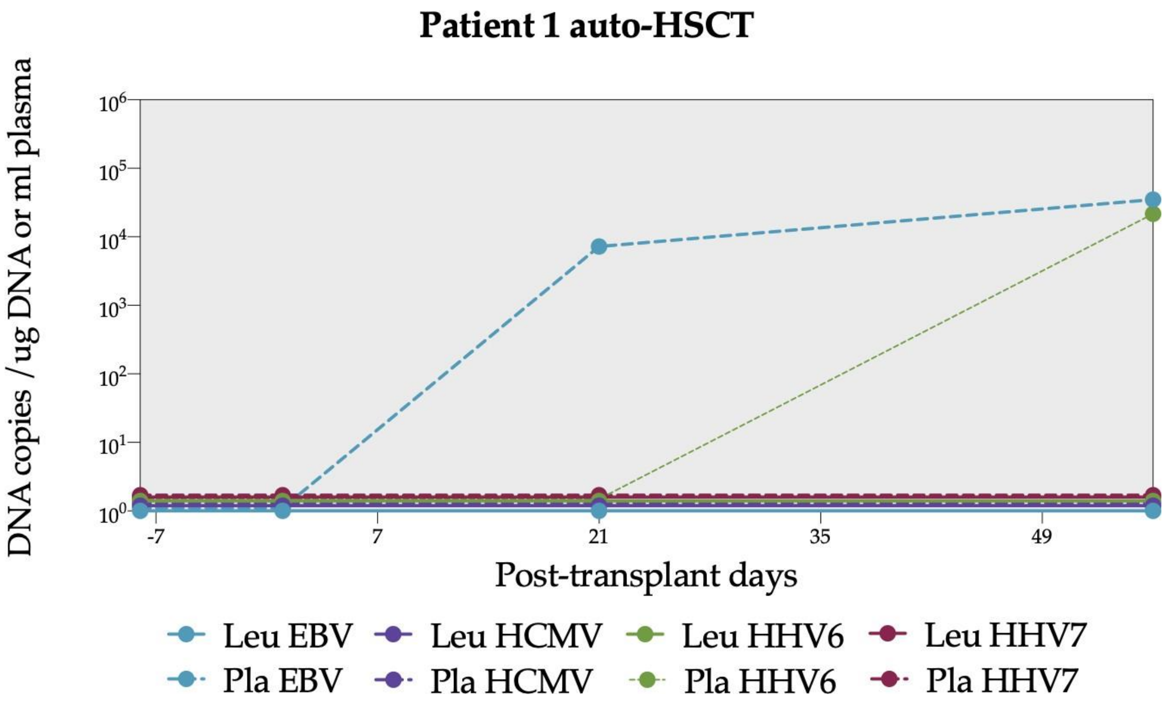

(A) Kinetics of infections in patient 1 who received autologous hematopoietic stem celltransplant (HSCT). The follow up is shown in the X axis and the viral load in the Y axis. Each virus is representedwith a specific color. Viral loads are expressed as DNA copies/ ug DNA in leukocytes or ml plasma.

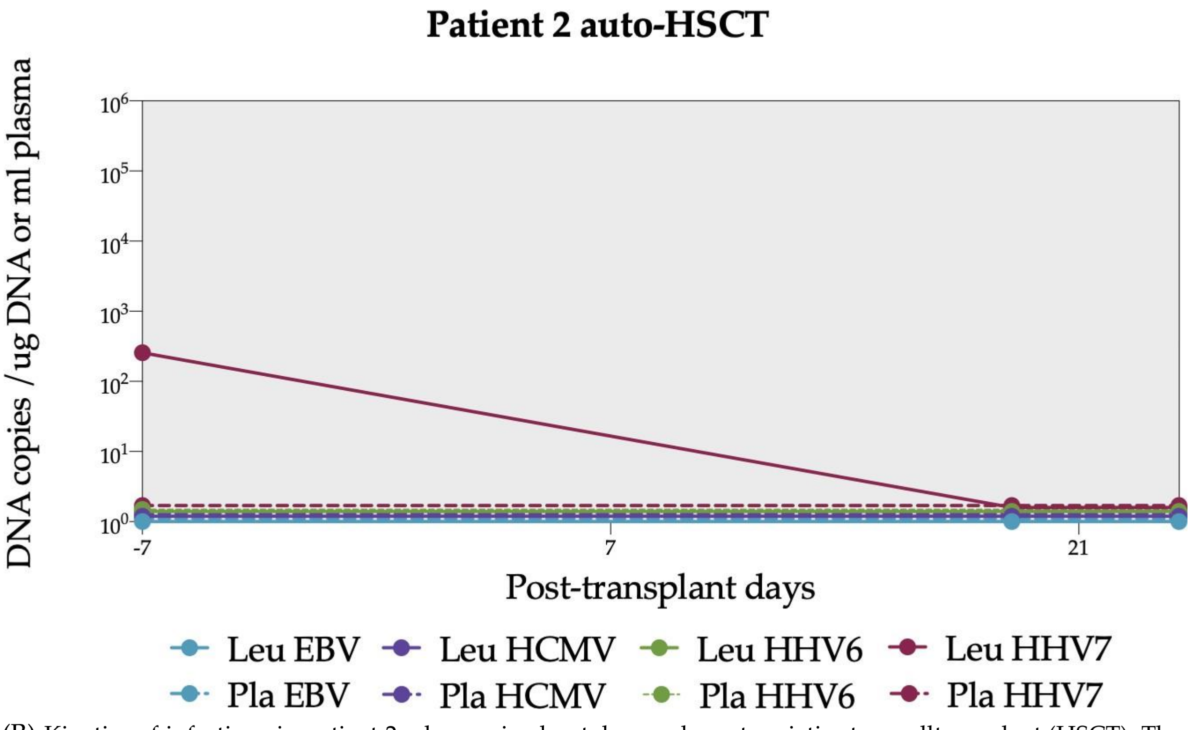

(B) Kinetics of infections in patient 2 who received autologous hematopoietic stem celltransplant (HSCT). The follow up is shown in the X axis and the viral load in the Y axis. Each virus is representedwith a specific color. Viral loads are expressed as DNA copies/ ug DNA in leukocytes or ml plasma.

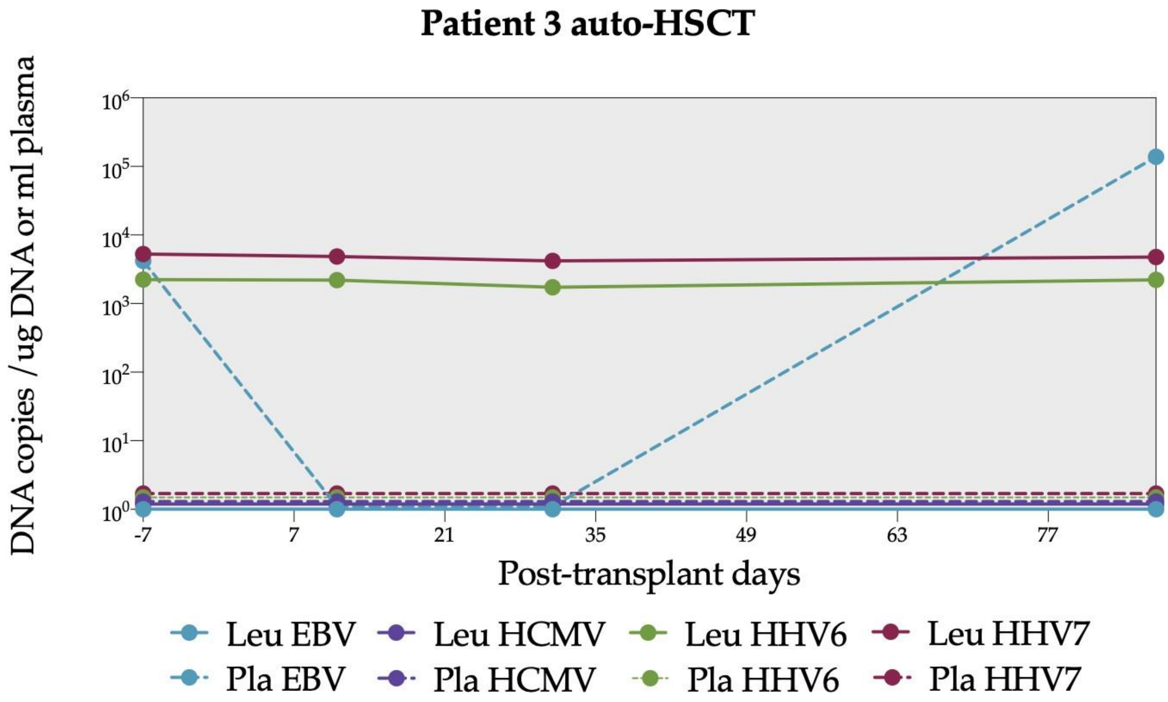

(C) Kinetics of infections in patient 3 who received autologous hematopoietic stem celltransplant (HSCT). The follow up is shown in the X axis and the viral load in the Y axis. Each virus is representedwith a specific color. Viral loads are expressed as DNA copies/ ug DNA in leukocytes or ml plasma.

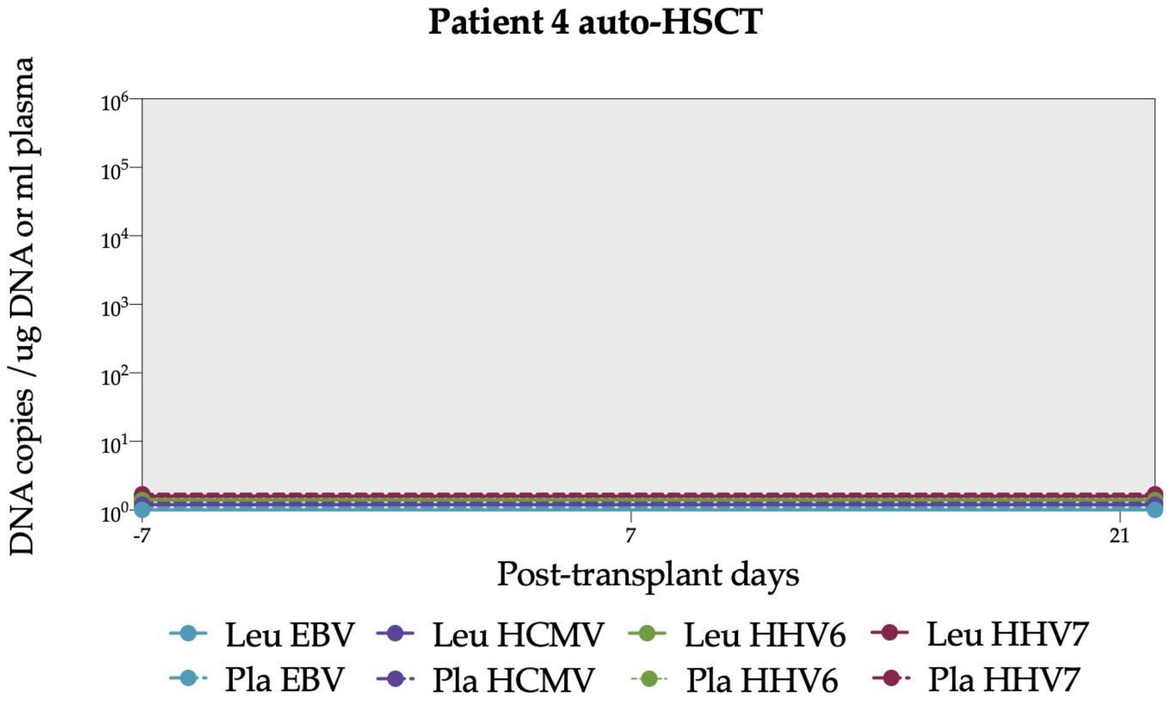

(D) Kinetics of infections in patient 4 who received autologous hematopoietic stem celltransplant (HSCT). The follow up is shown in the X axis and the viral load in the Y axis. Each virus is representedwith a specific color. Viral loads are expressed as DNA copies/ ug DNA in leukocytes or ml plasma

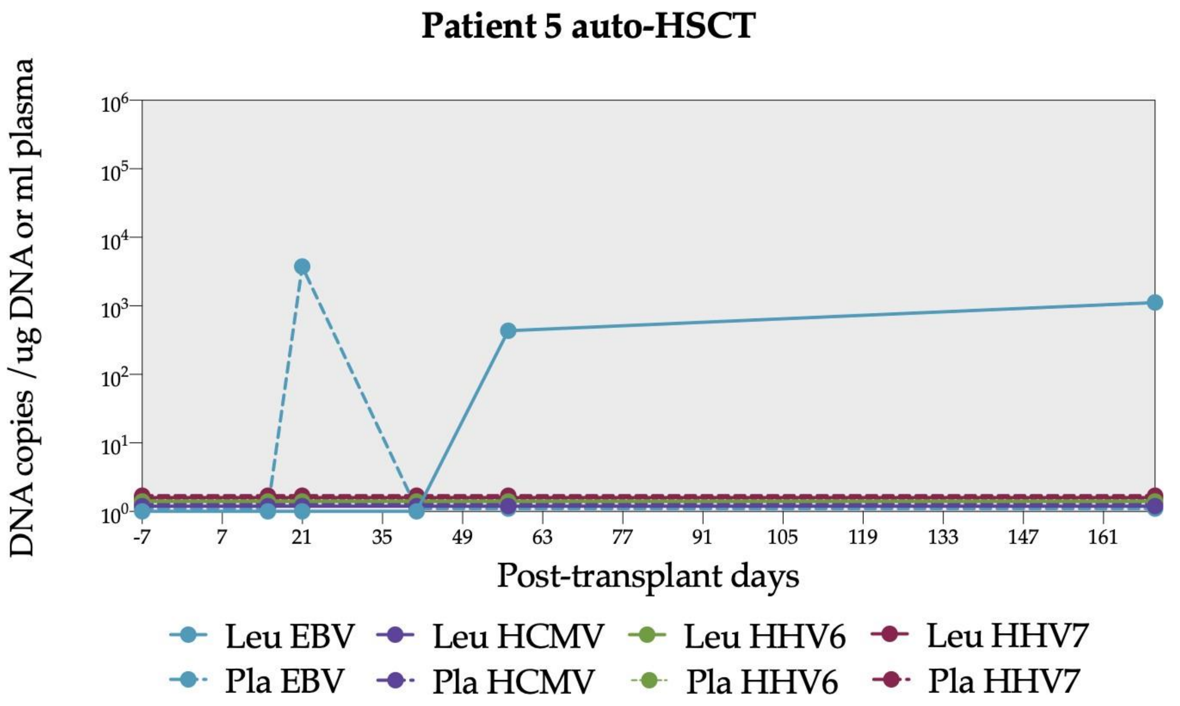

(E) Kinetics of infections in patient 5 who received autologous hematopoietic stem celltransplant (HSCT). The follow up is shown in the X axis and the viral load in the Y axis. Each virus is representedwith a specific color. Viral loads are expressed as DNA copies/ ug DNA in leukocytes or ml plasma.

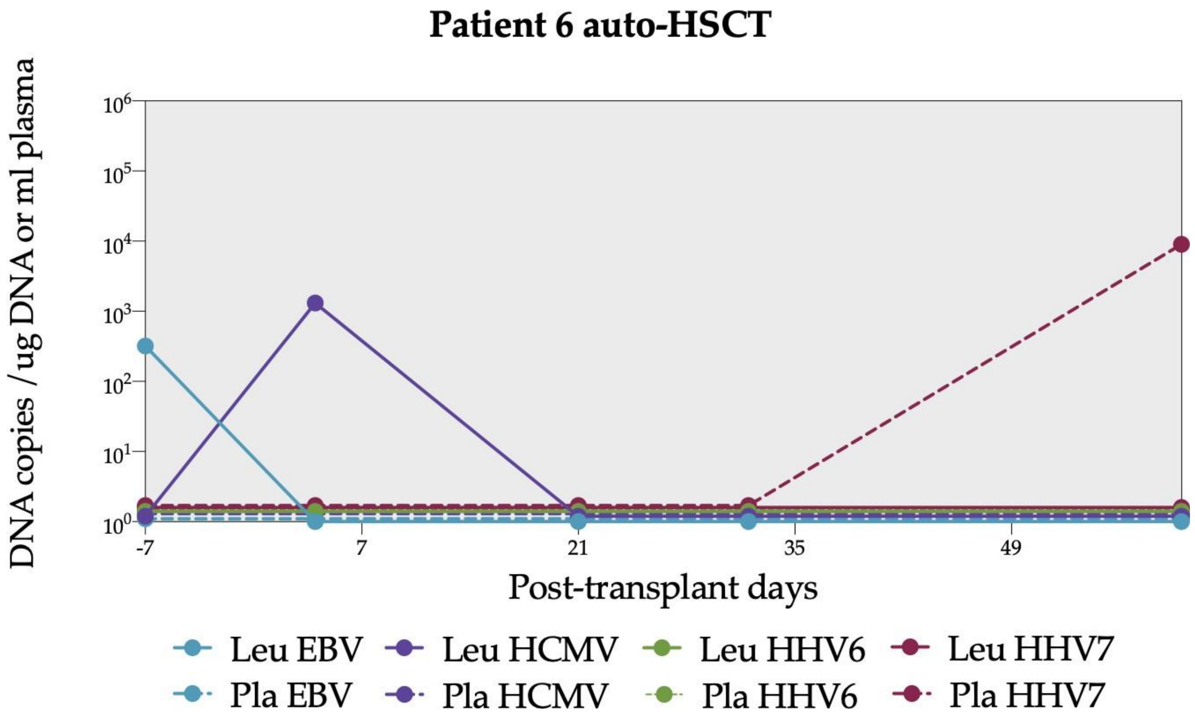

(F) Kinetics of infections in patient 6 who received autologous hematopoietic stem celltransplant (HSCT). The follow up is shown in the X axis and the viral load in the Y axis. Each virus is representedwith a specific color. Viral loads are expressed as DNA copies/ ug DNA in leukocytes or ml plasma.

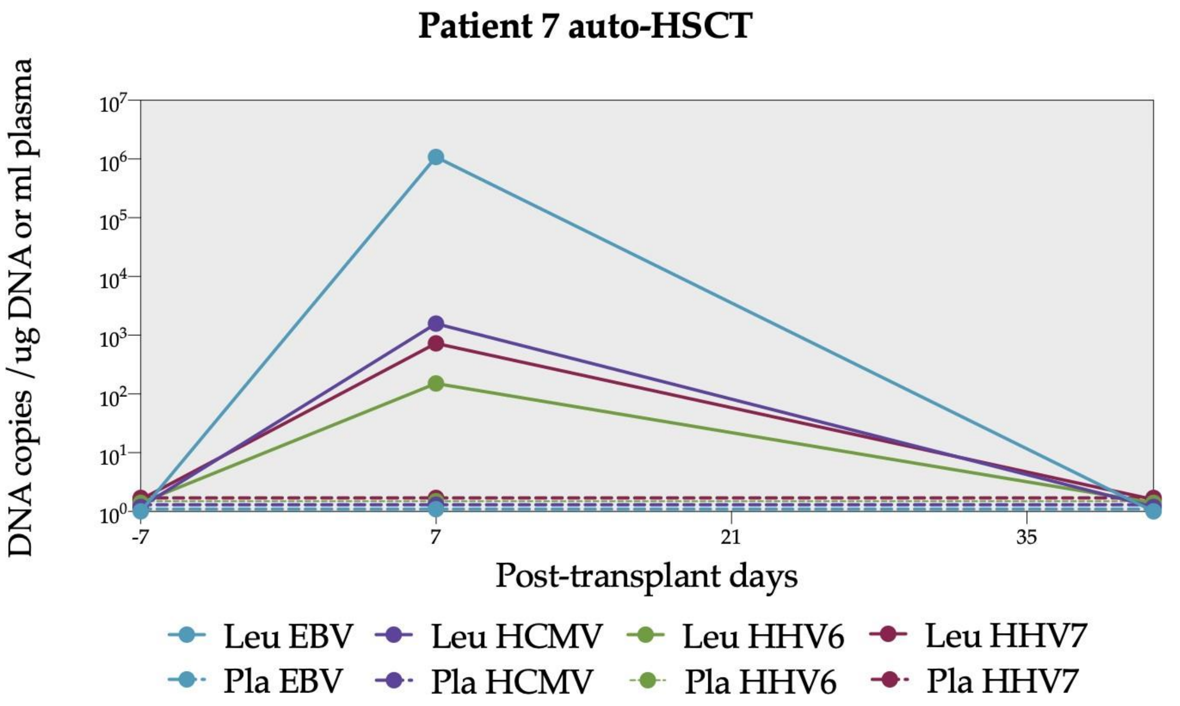

(G) Kinetics of infections in patient 7 who received autologous hematopoietic stem celltransplant (HSCT). The follow up is shown in the X axis and the viral load in the Y axis. Each virus is representedwith a specific color. Viral loads are expressed as DNA copies/ ug DNA in leukocytes or ml plasma.

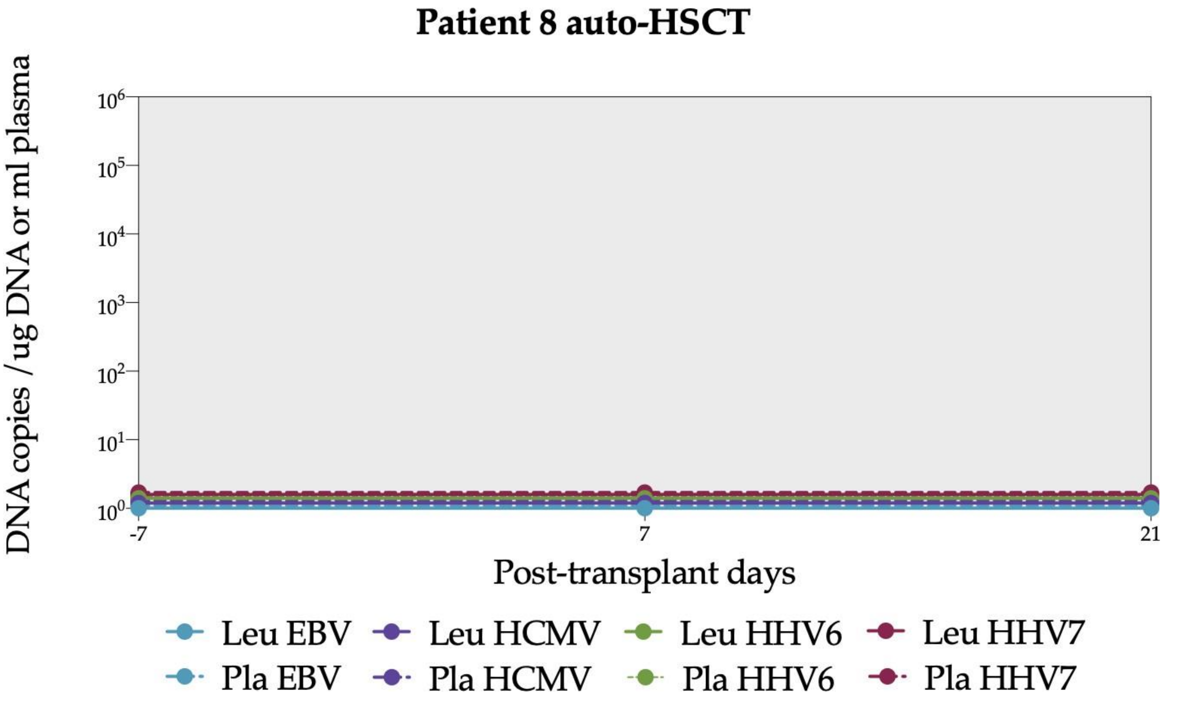

(H) Kinetics of infections in patient 8 who received autologous hematopoietic stem celltransplant (HSCT). The follow up is shown in the X axis and the viral load in the Y axis. Each virus is representedwith a specific color. Viral loads are expressed as DNA copies/ ug DNA in leukocytes or ml plasma.

Supplementary Figure S2. Viral loads for HCMV in patients with allogeneic and autologous hematopoietic stem cell transplantation (HSCT). Viral loads are expressed as DNA copies/ ug of DNA in leukocytes or ml of plasma. The red line indicates the clinical threshold used at the Children's Hospital of Mexico.

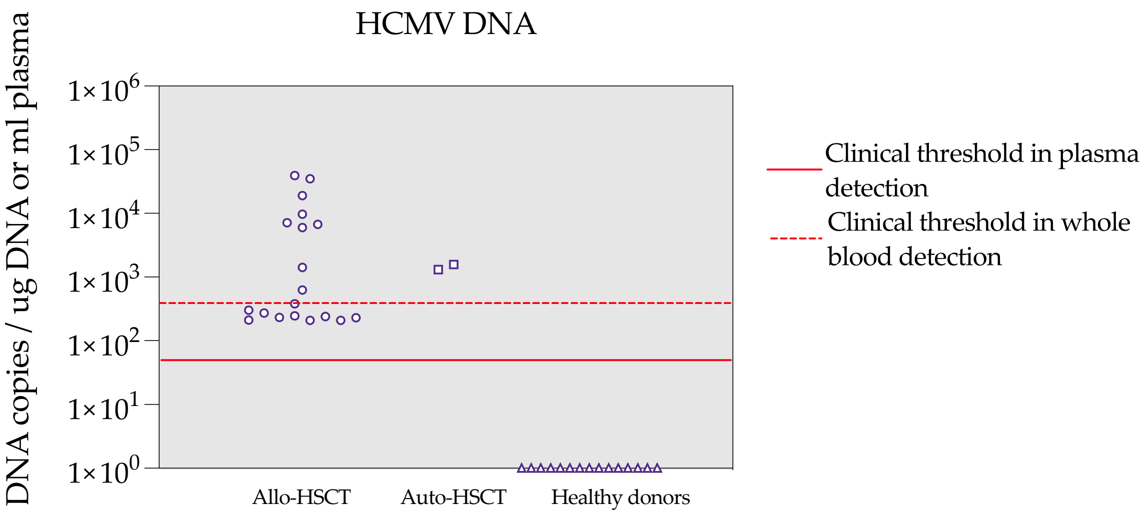

Supplementary Table S1. Pre-transplant serology for donors and recipients. Patients (n=40).

**Type of transplant**		**Pre-transplant EBV serology**		**Pre-transplant HCMV serology**	
		Donor	Recipient	Donor	Recipient
**Allogeneic (N=32)**	Patient 1 Allo-HSCT	Without data	IgG: + IgM: -	Without data	IgG: + IgM: -
	Patient 2 Allo-HSCT	Without data	Without data	Without data	IgG: + IgM: -
	Patient 3 Allo-HSCT	IgG: + IgM: -	IgG: + IgM: -	IgG: + IgM: -	IgG: + IgM: -
	Patient 4 Allo-HSCT	IgG: + IgM: -	IgG: + IgM: -	IgG: + IgM: -	IgG: + IgM: -
	Patient 5 Allo-HSCT	Without data	Without data	Without data	Without data
	Patient 6 Allo-HSCT	Without data	Without data	Without data	Without data
	Patient 7 Allo-HSCT	Without data	IgG: + IgM: -	Without data	IgG: + IgM: -
	Patient 8 Allo-HSCT	Without data	IgG: + IgM: -	Without data	IgG: + IgM: -
	Patient 9 Allo-HSCT	Without data	Without data	Without data	Without data
	Patient 10 Allo-HSCT	Without data	IgG: + IgM: -	Without data	IgG: + IgM: -
	Patient 11 Allo-HSCT	Without data	Without data	Without data	Without data
	Patient 12 Allo-HSCT	Without data	Without data	Without data	Without data
	Patient 13 Allo-HSCT	Without data	IgG: + IgM: -	Without data	IgG: + IgM: -
	Patient 14 Allo-HSCT	Without data	Without data	Without data	Without data
	Patient 15 Allo-HSCT	Without data	IgG: + IgM: -	Without data	IgG: + IgM: -
	Patient 16 Allo-HSCT	IgG: + IgM: -	IgG: + IgM: -	IgG: + IgM: -	IgG: + IgM: -
	Patient 17 Allo-HSCT	Without data	Without data	Without data	Without data
	Patient 18 Allo-HSCT	Without data	IgG: + IgM: -	Without data	IgG: + IgM: -
	Patient 19 Allo-HSCT	Without data	Without data	Without data	Without data
	Patient 20 Allo-HSCT	Without data	IgG: - IgM: -	Without data	IgG: + IgM: -
	Patient 21 Allo-HSCT	Without data	Without data	Without data	Without data
	Patient 22 Allo-HSCT	IgG: + IgM: -	IgG: - IgM: -	IgG: + IgM: -	IgG: - IgM: -
	Patient 23 Allo-HSCT	Without data	Without data	Without data	Without data
	Patient 24 Allo-HSCT	Without data	Without data	IgG: - IgM: -	Without data
	Patient 25 Allo-HSCT	Without data	IgG: + IgM: -	Without data	IgG: + IgM: -
	Patient 26 Allo-HSCT	Without data	Without data	Without data	Without data
	Patient 27 Allo-HSCT	Without data	IgG: + IgM: -	Without data	IgG: Ind IgM: -
	Patient 28 Allo-HSCT	IgG: + IgM: -	Without data	IgG: - IgM: -	Without data
	Patient 29 Allo-HSCT	Without data	Without data	Without data	Without data
	Patient 30 Allo-HSCT	Without data	IgG: + IgM: -	IgG: + IgM: -	IgG: + IgM: -
	Patient 31 Allo-HSCT	Without data	Without data	Without data	Without data
	Patient 32 Allo-HSCT	Without data	Without data	Without data	Without data
**Autologous (N=8)**	Patient 1 Auto-HSCT	IgG: - IgM: -		IgG: - IgM: -	
	Patient 2 Auto-HSCT	IgG: + IgM: -		IgG: - IgM: -	
	Patient 3 Auto-HSCT	IgG: + IgM: -		IgG: + IgM: -	
	Patient 4 Auto-HSCT	IgG: + IgM: -		IgG: + IgM: -	
	Patient 5 Auto-HSCT	IgG: + IgM: -		IgG: + IgM: -	

	Patient 6 Auto-HSCT	IgG: + IgM: -	IgG: + IgM: -
	Patient 7 Auto-HSCT	IgG: + IgM: -	IgG: + IgM: -
	Patient 8 Auto-HSCT	Without data	Without data

Ind means an indeterminate result. Patients who received autologous transplantation receive theirown stem cells.

Supplementary Table S2. Leukocyte counts in blood samples**.**

**Patient**	**Sample ID**	**Pre/Post transplant**	**Follow up days**	**Total of leukocytes (millions)**
**1 allo-HSCT**	1.00	Pre-transplant	-5	0.8
	1.03	Post-transplant	21	16.2
	1.04		35	19.4
	1.05		49	12.6
	1.06		68	16.5
	1.07		78	3.6
	1.09		110	2.1
	1.10		138	4.0
	1.12		160	13.8
	1.13		182	5.4
**2 allo-HSCT**	2.00	Post-transplant	24	1.2
	2.01		37	9.7
	2.02		52	6.5
	2.03		68	13.5
	2.04		85	8.2
	2.05		92	3.3
	2.06		106	3.2
	2.09		155	11.1
	2.10		162	8.6
	2.11		187	9.7
	2.13		207	12.7
	2.14		241	2.1
**3 allo-HSCT**	3.00	Pre-transplant	-7	8.5
	3.01	Post-transplant	7	11.3
	3.02		21	8.9
	3.03		30	0.7
	3.04		62	0.7
	3.07		86	1.2
**4 allo-HSCT**	4.00	Pre-transplant	-10	8.0
	4.02	Post-transplant	20	10.3
	4.05		74	7.7
	4.06		81	8.2
	4.07		113	3.6

	4.09		151	0.7
	4.10		165	2.5
	4.11		172	0.0
	4.12		193	2.9
	4.13		207	2.7
	4.15		280	2.3
	4.16		292	6.1
	4.17		330	4.9
	4.18		340	1.4
	4.19		351	2.8
**5 allo-HSCT**	6.00	Pre-transplant	-7	4.8
	6.02	Post-transplant	22	2.4
	6.04		48	2.9
	6.06		80	9.0
	6.07		94	1.4
	6.08		104	0.0
	6.09		132	4.6
	6.10		143	2.0
	6.11		157	2.0
	6.13		212	3.1
	6.14		233	1.1
	6.15		259	5.1
	6.16		294	1.0
	6.19		350	0.9
	6.20		364	2.8
**6 allo-HSCT**	9.00	Pre-transplant	-7	3.1
	9.02	Post-transplant	13	4.3
	9.03		21	5.2
	9.04		25	7.4
	9.05		75	7.4
	9.06		95	1.4
	9.07		106	2.5
	9.08		120	7.0
**7 allo-HSCT**	10.00	Pre-transplant	-8	0.2
	10.02	Post-transplant	21	7.8
	10.03		36	7.3
	10.04		49	7.3
	10.05		60	3.2
	10.06		88	6.9

	10.07		117	2.2
	10.09		152	3.4
	10.10		189	4.2
	10.11		202	0.7
	10.12		249	3.2
	10.13		277	2.2
	10.14		291	2.6
	10.15		305	2.1
	10.16		320	2.0
	10.17		344	3.2
**8 allo-HSCT**	12.00	Pre-transplant	-12	0.6
	12.02	Post-transplant	21	11.6
	12.03		33	16.1
	12.04		51	7.6
	12.05		65	7.9
	12.06		79	5.2
	12.07		89	4.5
	12.08		104	11.2
	12.09		110	7.5
	12.10		126	7.5
	12.11		138	3.4
	12.12		159	17.7
	12.13		173	10.4
	12.15		222	3.9
	12.16		245	11.9
	12.17		264	7.8
	12.18		279	19.9
	12.19		293	16.6
	12.20		314	1.8
	12.21		328	2.1
	12.22		342	2.5
**9 allo-HSCT**	13.00	Pre-transplant	-9	1.6
	13.02	Post-transplant	19	7.6
	13.03		32	2.7
	13.04		53	1.5
	13.05		68	1.8
	13.06		74	1.8
	13.07		95	2.0
	13.08		111	71.6

	13.09		131	13.3
	13.10		152	2.8
	13.11		168	9.6
	13.12		186	2.5
	13.13		202	5.5
	13.14		221	3.5
	13.15		236	2.5
	13.16		249	3.9
	13.17		271	2.6
	13.18		299	2.8
	13.19		319	5.4
**10 allo-HSCT**	14.00	Pre-transplant	-8	0.1
	14.02	Post-transplant	21	0.1
	14.03		31	11.4
	14.09		115	6.3
	14.10		136	0.4
	14.11		150	1.0
	14.12		168	2.2
	14.13		192	0.1
	14.14		206	2.8
	14.15		220	7.5
	14.16		235	0.1
	14.17		248	3.0
	14.18		270	0.1
	14.19		291	2.1
	14.20		327	3.9
**11 allo-HSCT**	15.01		7	0.1
	15.02		54	1.5
	15.03		65	3.1
	15.05		85	2.0
**12 allo-HSCT**	19.00	Pre-transplant	-7	5.3
	19.03	Post-transplant	35	3.5
	19.04		48	12.3
	19.05		61	2.3
	19.07		75	7.3
	19.09		121	1.4
	19.10		138	1.1
	19.11		152	4.8
	19.12		173	2.3

	19.13		187	2.4
	19.14		201	2.5
	19.15		236	3.6
**13 allo-HSCT**	20.00	Pre-transplant	-8	0.8
	20.03	Post-transplant	21	3.3
	20.05		55	1.2
	20.06		66	6.9
	20.07		76	2.3
**14 allo-HSCT**	22.00	Pre-transplant	-8	0.4
	22.01	Post-transplant	7	4.8
	22.02		26	9.1
	22.03		40	36.0
	22.04		46	1.7
	22.06		78	0.8
	22.08		102	4.1
	22.09		116	8.4
	22.10		130	5.9
	22.11		158	2.6
	22.12		172	44.8
	22.13		200	6.0
	22.14		215	0.9
	22.15		228	4.2
	22.16		252	21.8
**15 allo-HSCT**	23.00	Pre-transplant	-7	1.2
	23.01	Post-transplant	0	19.1
	23.03		21	17.2
	23.04		38	5.0
	23.05		56	1.8
**16 allo-HSCT**	24.00	Pre-transplant	-6	1.8
	24.01	Post-transplant	7	31.7
	24.02		21	3.1
	24.03		36	0.5
	24.04		50	20.1
	24.05		61	1.5
	24.06		75	1.7
	24.07		88	1.9
	24.08		102	5.9
**17 allo-HSCT**	25.00	Pre-transplant	-8	0.8
	25.02	Post-transplant	21	6.6

	25.03		33	0.8
	25.04		46	2.1
	25.05		61	2.6
	25.06		75	4.0
	25.07		95	12.6
	25.08		116	5.8
	25.09		131	4.2
	25.10		151	13.1
	25.11		183	5.1
	25.12		201	6.1
	25.13		214	3.8
	25.14		228	10.8
	25.15		242	5.1
	25.16		257	4.4
	25.17		271	7.0
	25.18		284	2.4
	25.19		299	3.1
	25.20		319	4.5
	25.21		334	4.4
**18 allo-HSCT**	26.00	Post-transplant	2	0.8
	26.02		30	4.0
	26.03		44	6.8
	26.04		62	1.9
	26.05		83	0.5
	26.06		96	2.9
	26.07		112	2.2
	26.08		132	5.4
	26.09		153	2.1
	26.10		170	3.5
	26.11		188	3.0
	26.12		202	0.8
	26.13		215	0.1
**19 allo-HSCT**	27.00	Pre-transplant	-7	5.8
	27.01	Post-transplant	11	0.4
	27.02		29	12.3
	27.03		41	6.4
	27.04		53	5.6
	27.05		67	3.7
	27.06		88	8.4

**20 allo-HSCT**	28.00	Pre-transplant	-7	6.4
	28.03	Post-transplant	48	40.0
	28.04		63	4.3
**21 allo-HSCT**	29.00		17	2.4
	29.02		21	5.6
	29.03		34	5.0
	29.04		48	0.6
	29.05		62	4.2
	29.06		76	13.4
	29.07		89	2.7
	29.08		104	1.3
	29.09		119	4.6
	29.10		132	2.7
	29.11		160	1.4
	29.12		174	3.7
	29.13		188	2.5
	29.14		202	2.5
	29.15		216	1.6
	29.16		230	3.4
	29.17		251	6.4
	29.18		272	3.6
	29.19		309	5.5
	29.20		351	3.5
**22 allo-HSCT**	30.00	Pre-transplant	-7	0.5
	30.01	Post-transplant	7	8.7
	30.02		21	11.8
	30.03		47	4.1
	30.04		61	3.2
	30.05		75	1.3
	30.06		90	6.8
	30.07		124	3.4
	30.08		133	3.4
	30.09		166	1.6
	30.10		180	1.7
	30.11		201	0.9
	30.12		236	3.2
	30.13		250	5.1
	30.14		272	5.5
	30.15		293	2.0

**23 allo-HSCT**	31.00	Pre-transplant	-2	0.2
	31.01	Post-transplant	14	2.2
	31.02		21	11.5
	31.03		35	4.7
	31.04		54	8.1
**24 allo-HSCT**	33.00	Pre-transplant	-9	3.0
	33.02	Post-transplant	21	9.7
	33.03		33	0.2
	33.04		47	0.5
	33.05		68	2.9
	33.06		89	2.9
**25 allo-HSCT**	34.00	Pre-transplant	-8	3.1
	34.02	Post-transplant	20	4.3
	34.03		36	3.5
	34.04		55	2.6
	34.05		69	2.0
	34.06		97	0.4
	34.07		112	1.2
	34.08		126	0.6
	34.09		146	0.5
	34.10		160	0.2
	34.11		191	2.7
	34.12		209	1.9
	34.13		223	1.2
	34.14		251	1.8
	34.15		322	1.5
	34.16		339	2.3
**26 allo-HSCT**	37.00	Post-transplant	6	1.4
	37.02		27	0.8
	37.03		46	1.1
	37.04		60	0.5
	37.05		82	0.3
	37.07		117	1.1
	37.08		131	2.0
	37.09		145	0.5
	37.10		159	3.3
	37.11		174	1.6
	37.12		187	5.3
	37.13		202	3.7

	37.14		215	0.7
	37.15		231	3.1
	37.16		287	0.6
**27 allo-HSCT**	40.00	Pre-transplant	-7	5.5
	40.03	Post-transplant	35	4.6
	40.04		49	1.1
	40.05		63	2.6
	40.06		77	0.4
	40.07		91	3.4
	40.08		104	2.0
	40.09		125	2.3
	40.10		181	1.3
	40.11		259	3.3
	40.12		293	6.0
	40.13		307	5.4
	40.14		323	1.9
	40.15		359	3.3
**28 allo-HSCT**	41.00	Pre-transplant	-7	3.5
	41.01	Post-transplant	4	0.3
	41.02		20	2.6
	41.03		40	5.6
	41.04		52	1.3
	41.05		68	3.3
	41.06		81	1.8
	41.07		95	1.4
	41.08		109	1.1
	41.09		171	3.6
	41.10		199	4.7
	41.11		227	3.8
	41.12		245	4.4
	41.13		292	7.0
	41.14		319	6.5
	41.15		332	4.3
	41.16		341	3.1
	41.17		363	16.1
**29 allo-HSCT**	42.00	Pre-transplant	-9	0.9
	42.02	Post-transplant	21	0.8
	42.03		34	2.9
	42.04		55	2.8

	42.05		69	3.7
**30 allo-HSCT**	44.00	Pre-transplant	-7	5.5
	44.02	Post-transplant	25	1.7
	44.03		35	1.8
	44.04		80	2.1
	44.05		101	3.8
	44.06		136	2.6
	44.07		171	7.5
	44.08		185	2.4
	44.09		199	4.2
	44.10		227	9.4
	44.11		238	5.0
**31 allo-HSCT**	45.00		0	3.3
	45.02		21	1.5
	45.03		35	0.2
	45.04		50	15.6
	45.05		61	15.0
	45.06		75	6.8
	45.07		85	1.5
	45.08		111	1.0
	45.09		132	2.3
	45.10		146	7.4
	45.11		169	0.8
	45.12		180	0.5
	45.13		202	1.2
	45.14		211	2.3
**32 allo-HSCT**	46.00	Pre-transplant	-4	0.5
	46.01	Post-transplant	7	0.5
	46.02		22	2.4
	46.03		34	1.8
	46.04		48	0.8
	46.05		63	4.0
	46.06		97	8.7
	46.07		111	4.2
	46.08		125	1.6
	46.09		134	0.1
	46.10		152	6.7
**1 auto-HSCT**	5.00	Pre-transplant	-8	5.4
	5.01	Post-transplant	1	1.3

	5.03		21	7.5
	5.04		56	5.7
**2 auto-HSCT**	7.00	Pre-transplant	-7	6.7
	7.02	Post-transplant	19	6.7
	7.03		24	1.2
**3 auto-HSCT**	8.00	Pre-transplant	-7	4.3
	8.03	Post-transplant	11	0.8
	8.04		31	16.3
	8.05		87	4.9
**4 auto-HSCT**	17.00	Pre-transplant	-7	1.6
	17.02	Post-transplant	22	9.5
**5 auto-HSCT**	32.00	Pre-transplant	-7	1.0
	32.01	Post-transplant	15	0.8
	32.02		21	1.8
	32.03		41	0.6
	32.04		57	8.7
	32.05		170	12.9
**6 auto-HSCT**	35.00	Pre-transplant	-7	6.0
	35.01	Post-transplant	4	1.1
	35.02		21	2.4
	35.03		32	2.4
	35.04		60	7.6
**7 auto-HSCT**	38.00	Pre-transplant	-7	0.6
	38.01	Post-transplant	7	5.5
	38.02		41	5.5
**8 auto-HSCT**	43.00	Pre-transplant	-7	1.7
	43.01	Post-transplant	7	1.8
	43.02		21	2.5
